# A Growth-Based Framework for Leaf Shape Development and Diversity

**DOI:** 10.1016/j.cell.2019.05.011

**Published:** 2019-05-30

**Authors:** Daniel Kierzkowski, Adam Runions, Francesco Vuolo, Sören Strauss, Rena Lymbouridou, Anne-Lise Routier-Kierzkowska, David Wilson-Sánchez, Hannah Jenke, Carla Galinha, Gabriella Mosca, Zhongjuan Zhang, Claudia Canales, Raffaele Dello Ioio, Peter Huijser, Richard S. Smith, Miltos Tsiantis

**Affiliations:** 1 Department of Comparative Development and Genetics, Max Planck Institute for Plant Breeding Research, Carl-von-Linné-Weg 10, 50829, Cologne, Germany; 2 Department of Plant Sciences, University of Oxford, South Parks Road, Oxford OX1 3RB, UK

**Keywords:** leaf development, growth and patterning, live-imaging, organ shape, *Cardamine hirsuta*, *Arabidopsis thaliana*, *KNOX*, *RCO*, computational modelling, morphogenesis

## Abstract

How do genes modify cellular growth to create morphological diversity? We study this problem in two related plants with differently shaped leaves: *Arabidopsis thaliana* (simple leaf shape) and *Cardamine hirsuta* (complex shape with leaflets). We use live imaging, modeling, and genetics to deconstruct these organ-level differences into their cell-level constituents: growth amount, direction, and differentiation. We show that leaf shape depends on the interplay of two growth modes: a conserved organ-wide growth mode that reflects differentiation; and a local, directional mode that involves the patterning of growth foci along the leaf edge. Shape diversity results from the distinct effects of two homeobox genes on these growth modes: *SHOOTMERISTEMLESS* broadens organ-wide growth relative to edge-patterning, enabling leaflet emergence, while *REDUCED COMPLEXITY* inhibits growth locally around emerging leaflets, accentuating shape differences created by patterning. We demonstrate the predictivity of our findings by reconstructing key features of *C. hirsuta* leaf morphology in *A. thaliana.*

**Video Abstract:**

## Highlights

• Complete growth and fate maps are made for *C. hirsuta* and *A. thaliana* leaf surfaces
• Patterning of the leaf margin modifies organ-wide growth pattern to produce leaf shape
• Altering growth relative to patterning generates leaf shape diversity
• Reconstructing dissected leaf shape by combining STM and RCO in *A. thaliana* leaves

## Graphical Abstract

**Figure:**
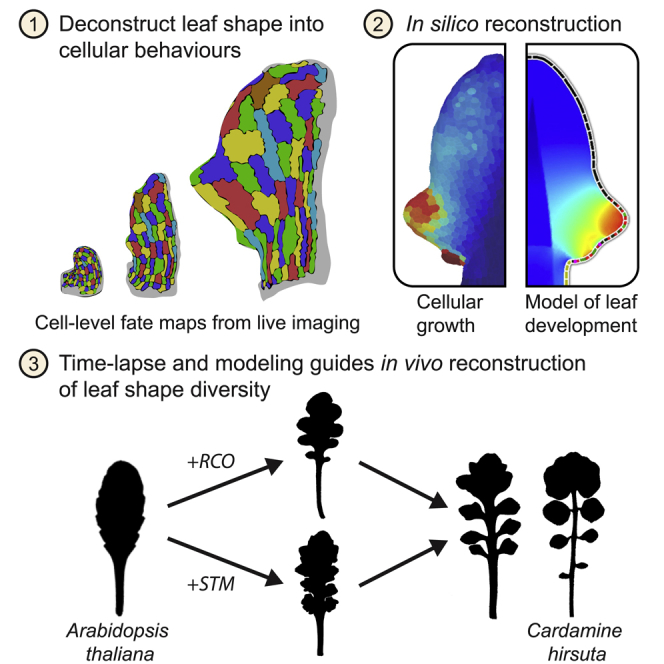

## Teaser

By understanding the impact of growth and patterning on leaf shape diversity, it is possible to convert the leaf morphology of *A. thaliana* to that of another plant species.

## Introduction

How gene activity translates into distinct organ morphologies remains poorly understood (e.g., Runions and Tsiantis, 2017, Zuniga, 2015). To understand the complex interactions that link gene action to tissue form, we need quantitative data on cellular growth at high spatial and temporal resolution, which are technically challenging to acquire (Etournay et al., 2016, Fox et al., 2018). Such data are also difficult to interpret because local gene activity can have complex non-local effects on tissue deformations, which arise from interactions between genetically specified growth and tissue mechanics (Coen and Rebocho, 2016). We also do not yet understand how evolutionary changes in gene activity alter how growth, patterning, and differentiation interact to produce diverse organ forms.

**Figure 1:**
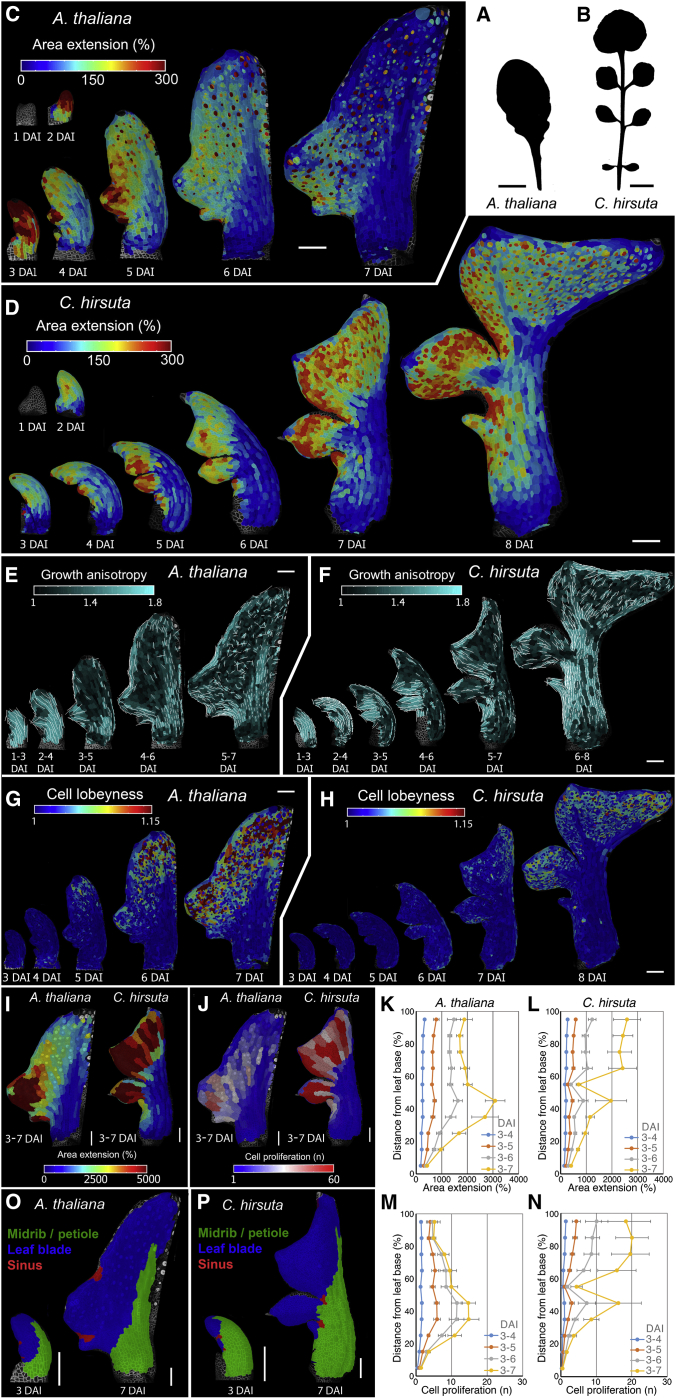
Conserved versus Divergent Growth Patterns in *A. thaliana* and *C. hirsuta* Leaves (A and B) Eighth rosette leaf of *A. thaliana* (A) and *C. hirsuta* (B). (C–H) Heat-maps of area extension (C and D), growth anisotropy (E and F), and cell lobeyness (G and H) for *A. thaliana* (C, E, and G) and *C. hirsuta* (D, F, and H) leaves. Lobeyness measures pavement cell undulation (Sapala et al., 2018, see STAR Methods). White lines in (E) and (F) indicate cell-growth orientation where anisotropy > 40%. In both species, proliferation and growth increased at protrusions and decreased in adjacent sinuses (Figure S2). At the leaf margin, a basipetal transition from dynamic growth to tissue-dependent patterning occurs, coinciding with differentiation progression (C–H). See also Figures S1A–S1J. (I and J) Heat-maps of area extension (I) and cell proliferation (J), 3–7 DAI for *A. thaliana* (left) and *C. hirsuta* (right). (K–N) Growth alignment graph of mean area extension (K and L), and cell proliferation (M and N), from 3–7 DAI as a function of distance from leaf base in *A. thaliana* (K and M) and *C. hirsuta* (L and N). Error bars, SEM (n = 5–28, K and M; n = 11–27, L and N). (O and P) Lineage tracing of leaf blades (blue), petiole and midrib (green), and sinuses (red) in *A. thaliana* (O) and *C. hirsuta* (P). DAI indicates days after primordium initiation. Scale bars, 1 cm in (A) and (B), 100 μm in (C)–(J), (O), and (P). See also Figure S1 and S2 and Video S1. Replication for imaging data is reported for all figures in STAR Methods.

Plant leaves are an attractive system in which to address these questions as they grow from almost indistinguishable primordia into shapes that vary tremendously among species. Leaves can be simple, with smooth undivided margins (the botanical term for leaf edge), or complex, with protrusions of different size and geometry. The leaves of *A. thaliana* are simple and bear small marginal protrusions called serrations, while those of its relative, *C. hirsuta*, are dissected into distinct leaflets that each resemble a simple leaf (Figures 1A and 1B). Genetic studies have identified *KNOX* (*Knotted1*-like homeobox) and *RCO* (*REDUCED COMPLEXITY*) homeobox genes as important molecular regulators of leaf complexity that are involved in the evolutionary diversification of leaf form (Bharathan et al., 2002, Hareven et al., 1996, Hay and Tsiantis, 2010, Vlad et al., 2014). While target genes are known for some KNOX proteins (Bolduc et al., 2012), we still do not understand how *KNOX* and *RCO* affect cell- and tissue-level growth during leaf primordium development. Furthermore, we lack information on the growth of morphogenetically important domains at the margin and base of developing leaves, and cell-level fate maps for leaf primordia do not exist. It thus remains unclear how local growth regulation at the margin integrates with global patterns of growth, proliferation, and differentiation to produce divergent leaf forms (Bar and Ori, 2014, Bilsborough et al., 2011, Alvarez et al., 2016, Donnelly et al., 1999, Fox et al., 2018, Kuchen et al., 2012, Poethig, 1987). For example, current evidence indicates that growth polarity is vital for leaf geometry, yet the degree to which this polarity is a local or global feature of organ development and how it shapes leaf form remain unclear (Bringmann and Bergmann, 2017, Kuchen et al., 2012, Mansfield et al., 2018). Computational modeling offers one way to address these questions (Ali et al., 2014), by enabling us to investigate how multiple processes interact to create geometry in a growing tissue.

Here, we identify differences in growth patterns that yield simple, elliptical *A. thaliana* leaves versus dissected *C. hirsuta* leaves with a broad terminal leaflet. We use live imaging and genetics alongside computational modeling and analyses of growth to deconstruct these two divergent leaf forms into their cell-level constituent elements: the amount and direction of growth and the rate of differentiation. Surprisingly, we find that key features of growth are conserved between these two leaf forms. Differences in leaf geometry originate from two distinct processes that act in *C. hirsuta*, but not in *A. thaliana* leaves. In the first process, which requires the *KNOX* gene *SHOOTMERISTEMLESS* (*STM*), delayed differentiation and slower but prolonged growth throughout the leaf primordium increase the size and number of protrusions initiated by a conserved auxin-based pattern-generating mechanism. This process also allows protrusions to grow for longer in a polarized fashion. In the second process, local growth inhibition, mediated by the *RCO* gene, accentuates growth differences created by marginal patterning. We demonstrate the predictive power of our approach by experimentally reconstructing key aspects of the dissected *C. hirsuta* leaf form in *A. thaliana*.

## Results

### Development of Simple versus Dissected Leaf Primordia

We developed an imaging protocol (see STAR Methods) to understand how the balance of conserved versus diverged cellular growth patterns produce the simple leaf forms of *A. thaliana* and the dissected leaves of *C. hirsuta* (Figures 1A and 1B). We measured leaf primordium growth at cellular resolution, from its emergence until 7–8 days after initiation (DAI), when shape divergence between the two species is established. Our measurements included the leaf margin where serrations and leaflets form (Figure 1; Video S1). We computed complete lineage maps from these data (Barbier de Reuille et al., 2015) to understand how cells in the early primordium contribute to the development of the mature leaf form. Using the lineage maps, we quantified cell growth parameters that affect form (see STAR Methods), including growth amount (rate of cell-area increase), directionality (anisotropy, the ratio of expansion in the max. and min. principal directions of growth), and cell proliferation. To assess the progression of tissue differentiation, we also measured cell size, stomatal density, and pavement cell geometry.

Video S1. Growth of Wild-Type A. thaliana and C. hirsuta Leaves, Related to Figure 1

**Figure S1:**
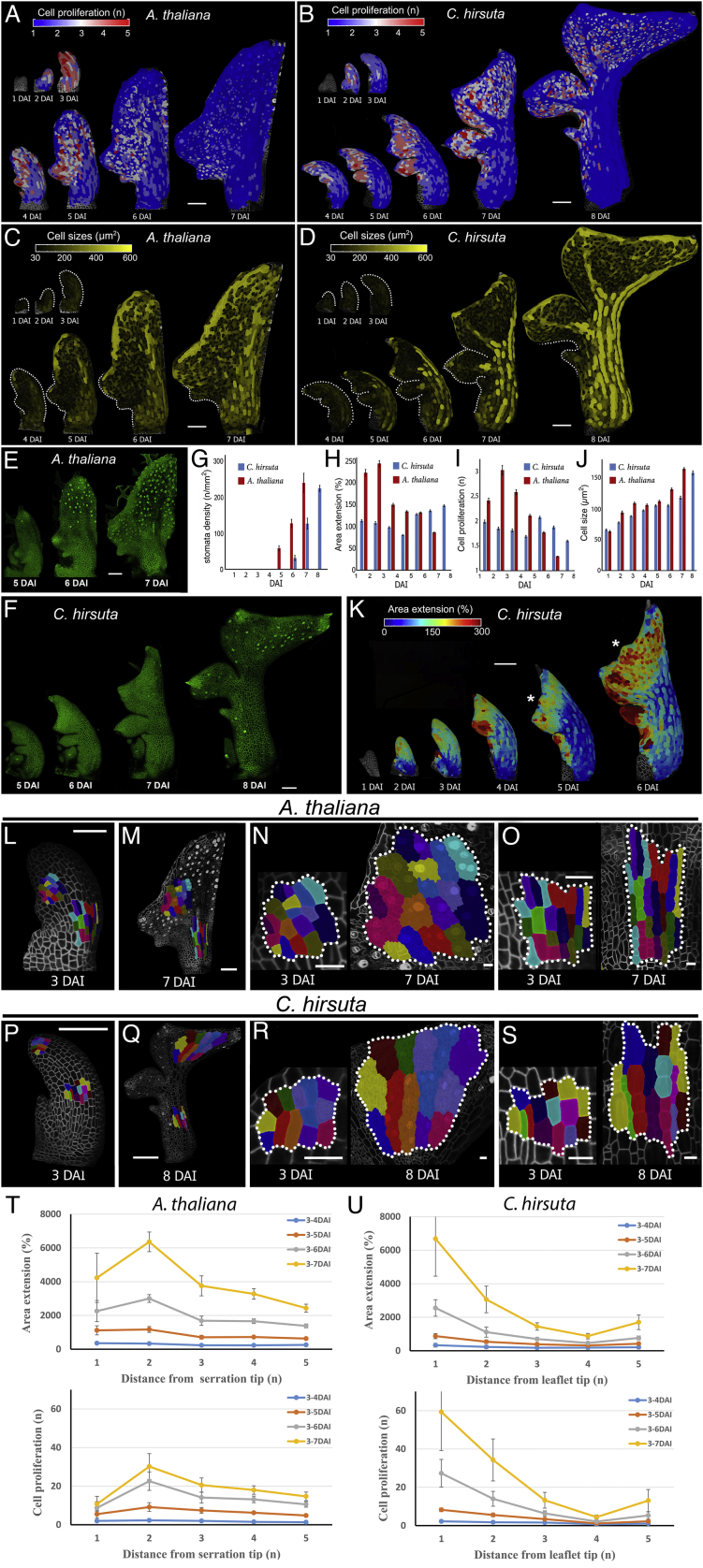
Analysis of the Conserved versus Divergent Aspects of Growth of *A. thaliana* and *C. hirsuta* Leaves, Related to Figure 1 (A-D) Heat-maps of cell proliferation (A-B) and cell size (C-D) for *A. thaliana* (A,C), and *C. hirsuta* (B,D) leaves. (E-F) Confocal time-lapse series of developing *A. thaliana* (E) and *C. hirsuta* (F) leaves with plasma membrane marker in green. Note the earlier appearance of stomata (dots marked with stronger YFP signal) in *A. thaliana*. (G) Bar-graph representing stomata density in relation to DAI (error bars indicate SE). n = 2-7 (*A. thaliana*), n = 3-6 (*C. hirsuta*). (H-J) Quantifications of mean area extension (H), cell proliferation (I), and cell size (J) for *A. thaliana* and *C. hirsuta* leaves (error bars indicate SE). *A. thaliana,* n > 100 (3 independent time-lapse series); *C. hirsuta*, n > 350 (4 independent time-lapse series). (K) Heat-maps of area extension for a *C. hirsuta* leaf. Asterisk indicates additional protrusion emerging from the leaf margin of the terminal leaflet. (L-S) Cell lineage tracing analysis in the leaf blade (N and R) or the petiole/midrib (O and S) for *A. thaliana* (L-O) and *C. hirsuta* (P-S) leaves. Location of the sectors at the beginning (L and P) and end (M and Q) of observations. Colors show the correspondence between cells at 3 DAI and their clonal sectors at 7 DAI. (T-U) Growth alignment graphs showing mean area extension and cell proliferation from 3-7 DAI as a function of distance from the tip of the first serration in *A. thaliana* (T) and leaflet in *C. hirsuta* (U). Distance is measured in cell-number. Error bars indicate SEM (n = 5-28, T; n = 3-24, U). DAI indicates days after primordia initiation. Scale bars, 100 μm in (A-F, K-M, P-Q), and 20 μm in (N-O and R-S).

**Figure S2:**
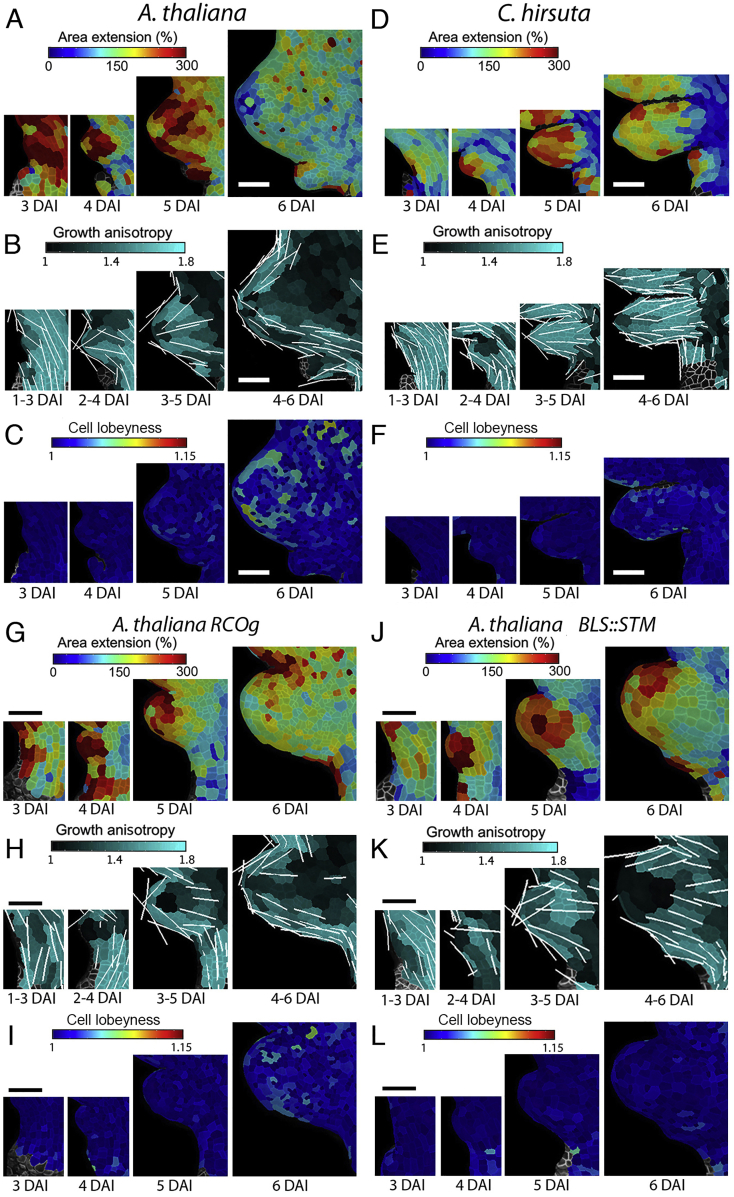
Growth Patterns of Margin Protrusions, Related to Figure 1, Figure 2, Figure 3, Figure 4 (A-C) Heat-maps of area extension (A), growth anisotropy (B), and cell lobeyness (C) for *A. thaliana* WT serration. (D-F) Heat-maps of area extension (D), growth anisotropy (E), and cell lobeyness (F) for *C. hirsuta* WT lateral leaflet. (G-I) Heat-maps of area extension (G), growth anisotropy (H), and cell lobeyness (I) for *A. thaliana RCOg* lobe. (J-L) Heat-maps of area extension (J), growth anisotropy (K), and cell lobeyness (L) for *A. thaliana BLS::STM* lobe. White lines in (B, E, H and K) indicate the orientation of cell growth where anisotropy is higher than 40%. Scale bars 50 μm.

We observed that despite their different final forms, leaves from these two species shared three commonalities in their growth patterns (Figures 1C–1H and S1A–S1S). (1) During leaf initiation (1–3 DAI), primordium growth is uniform and anisotropic. (2) After the initiation phase (∼3–4 DAI), dynamic marginal growth patterns are established in lateral regions, which are defined by changing patterns of proliferation, growth, and anisotropy that accompany protrusion outgrowth (Figures 1C–1F and S2A–S2F). (3) Later, in association with the basipetal (i.e., tip-to-base) progression of differentiation, tissue-dependent growth patterns emerge, with mainly isotropic growth occurring in the blade and anisotropic growth occurring along the margin circumference, midrib, and petiole (Figures 1E, 1H and S1L–S1S). Notably, in both plants, fronts of differentiation initiated at the leaf tip and each protrusion tip, rather than as a single front, and progressed toward the leaf base (Figures 1C–1D, 1G, 1H, S1A–S1D, and S2A–S2F). Proliferation decreased abruptly in the midrib/petiole region at ∼4 DAI (Figures S1A–S1D) with differentiation onset, while in the rest of the blade, proliferation decreased gradually, coinciding with the basipetal progression of differentiation. These results indicate that species-specific differences in leaf form emerge in the context of these three conserved growth patterns.

To identify in an unbiased manner the differences in cellular growth distribution that yield these distinct leaf forms, we quantified and compared the growth and proliferation of clonal lineages of each species from 3–7 DAI (Figures 1I and 1J). In this context, we also computed growth alignment graphs by mapping growth and proliferation, according to the location of cells along a leaf’s Proximal-Distal axis (P-D axis, location assessed at 3 DAI, Figures 1K–1N). This approach provides a developmental biology equivalent of a sequence alignment, and we used it to determine the balance of conservation versus divergence in cell-level growth properties at a given developmental stage in diverse genotypes. Although primordium length was comparable between species at both 3 and 7 DAI (∼150 and ∼800 μm, respectively), the P-D distributions of growth and proliferation differed (Figures 1I–1N). Growth and proliferation were restricted to proximal regions in *A. thaliana* but were broadly distributed in the *C. hirsuta* blade (Figures 1I and 1J), increasing the contribution of the distal primordium to the leaf surface (Figures 1K–1N) and correlating with the emergence of a broad terminal leaflet. Increased distal growth in *C. hirsuta*, compared to *A. thaliana*, coincided with the delayed onset of tissue-dependent growth patterns (Figures 1C–1H), delayed differentiation (Figures S1E–S1J), and extended dynamic marginal growth (as indicated by the emergence of additional protrusions in the terminal leaflet, Figure S1K). Thus, the distribution of growth and proliferation in *C. hirsuta* leaves is shifted distally along the P-D axis relative to *A. thaliana*. This shift is associated with prolonged marginal patterning and a global delay in differentiation.

We next investigated the local growth features that underlie the different leaf protrusion shapes of each species. Protrusion shape is influenced by the growth differential between rapidly growing protrusion tips and slow-growing adjacent tissues (Bilsborough et al., 2011, Nikovics et al., 2006, Vlad et al., 2014). We observed large differences in this growth differential between species (Figures S1T and S1U). Compared to serrations, the duration of anisotropic growth was extended in leaflets (2–6 DAI in leaflets versus 2–5 DAI in serrations; Figures 1E–1F, S2B, and S2E), as was the duration of reduced growth and proliferation in the sinus regions between leaflets (3–7 DAI in leaflets versus 3–5 DAI in serrations, Figure 1C, 1D, S1A, S1B, S2A, and S2D). Consequently, in *C. hirsuta*, both increased protrusion outgrowth and reduced growth at the protrusion base contributed to leaflet formation (compare Figure S1T and S1U). Reduced growth at the protrusion base likely reflects local growth inhibition (Vlad et al., 2014) but may also result from global differences in the growth of primordia (*C. hirsuta* growth is almost half that of *A. thaliana* over 1–3 DAI, Figures S1H and S1I). Thus, reduced growth at the protrusion base distinguishes leaflets in *C. hirsuta* from serrations in *A. thaliana* and likely involves both local and global growth regulation.

The hypothesis that both global and local aspects of growth contribute to the increased growth inhibition between protrusions in *C. hirsuta* leaves raised the question of how these two different growth modes interact to shape leaf geometry. To investigate this, we examined the origin of protrusions and sinuses relative to the global (organ-wide) growth patterns of the leaf blade, petiole, and midrib. Based on patterns of growth, proliferation, and cell morphology (Figures 1C–1J and S1A–S1D), we divided 3 and 7 DAI primordia into three regions: (1) blade, (2) petiole and midrib, and (3) the slowly growing sinus cells that lie adjacent to emerging protrusions (Figures 1O–1P, see STAR Methods). We found that in *A. thaliana*, sinuses initiated in the leaf blade and were thus always surrounded by rapidly growing blade cells (Figure 1O). By contrast, at 3 DAI in *C. hirsuta*, growth-repressed zones appeared directly adjacent to the midrib (Figure 1P), preventing blade establishment between protrusions and enabling the formation of a dissected leaf. Thus, sinus establishment in the blade is associated with a simple leaf, and in the midrib, with a dissected leaf.

These results suggest that three key differences distinguish dissected leaves from simple ones: (1) the local context in which protrusions initiate (midrib versus blade); (2) decreased growth at the base of initiating marginal protrusions; and (3) a global change in growth pattern, coinciding with delayed differentiation and marked by increased lateral and distal growth and by reduced growth in proximal and medial regions. Thus, both global and local factors influence growth to shape simple and dissected leaves.

### Relationship between Marginal Patterning and Leaf Growth

**Figure 2:**
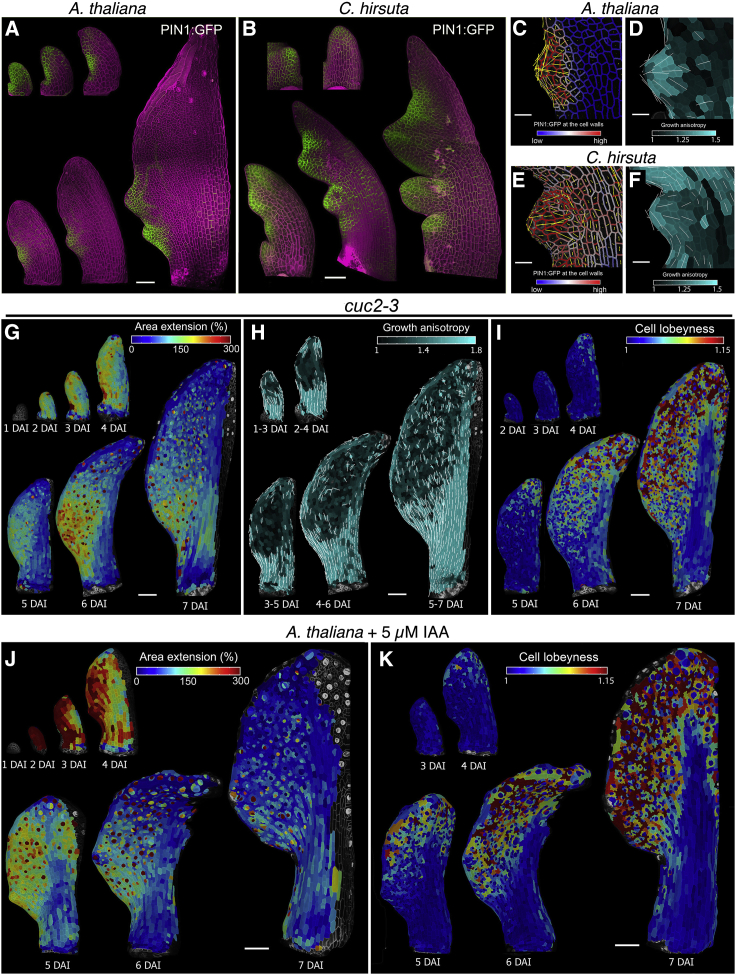
Relationship between Marginal Patterning and Growth (A and B) Localization of *pPIN1::PIN1:GFP* (green) in *A. thaliana* (A) and *C. hirsuta* (B) leaves at consecutive developmental stages. (C–F) *pPIN1::PIN1:GFP* signal distribution near cell border (C and E) and growth anisotropy (D and F) in *A. thaliana* (C and D) and *C. hirsuta* (E and F) emerging protrusions. (G–I) Heat-maps of area extension (G), growth anisotropy (H), and cell lobeyness (I) for *cuc2-3 A. thaliana* mutant. (J and K) Heat-maps of area extension (J) and cell lobeyness (K) for 5 μm IAA treated *A. thaliana* leaves. Yellow lines in (C) and (E) indicate cellular orientation of PIN1 polarization. White lines in (D), (F), and (H) indicate cell growth orientation when anisotropy > 20% (D and F) or 40% (H). Scale bars, 100 μm (A and B, G–K), 20 μm (C–F). See also Figure S2, Figure S3, Figure S4, Figure S5 and Video S2.

**Figure S3:**
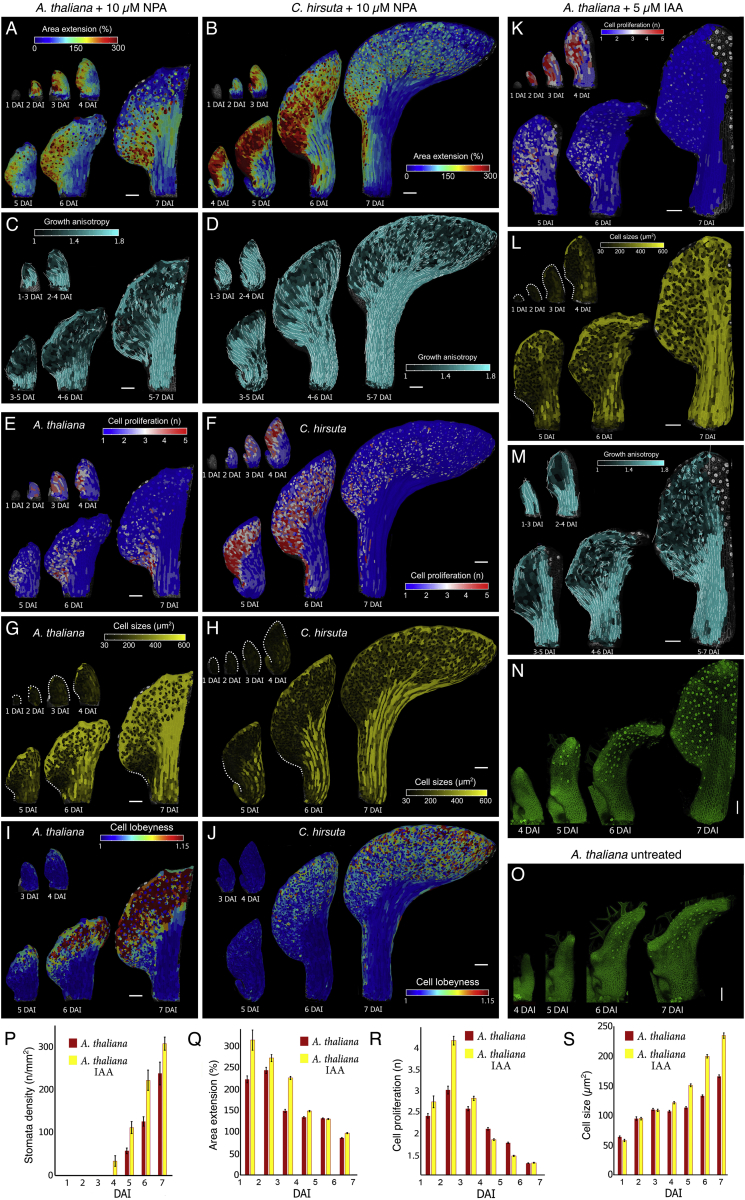
IAA and NPA Treated Wild-Type *A. thaliana* Leaves, Related to Figure 2 (A-J) Heat-maps of area extension (A,B), growth anisotropy (C,D), proliferation (E,F), cell size (G,H) and lobeyness (I,J) for NPA-treated *A. thaliana* (A,C,E,G,I), and *C. hirsuta* (B,D,F,H,J) leaves. (K-M) Heat-maps of cell proliferation (K), cell size (L), and growth anisotropy (M) for a *A. thaliana* wild-type leaf treated with 5 μM IAA. (N-O) Confocal time-lapse series of the developing *A. thaliana* leaf treated with 5 μM IAA (N) as compared to the control (O). (P-S) Quantifications of stomatal density (P), mean area extension (Q), cell proliferation (R), and cell size (S) for *A. thaliana* WT leaves treated with 5 μM IAA as compared to control (error bars indicate SEM). *A. thaliana* leaf treated with 5 μM IAA, n = 3-7 (P) and n > 80 (Q-S, 4 independent time-lapse series). White lines in (C-D, M) indicate cell growth orientation where anisotropy > 40%. Scale bars, 100 μm. Dotted lines in (L) indicate leaf outlines.

**Figure S4:**
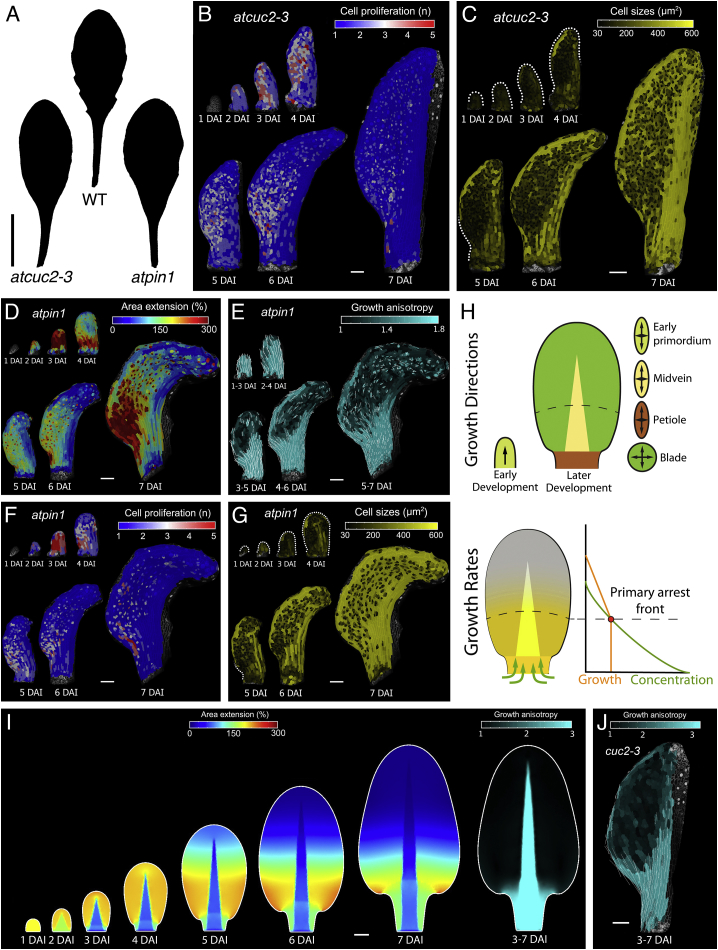
Leaf Growth Characteristics Converge in the Absence of Marginal Patterning, Related to Figures 2 and 3 (A) Silhouettes of *A. thaliana* wild-type (middle), *cuc2-3* (left), and *pin1* (right) mutant leaves. (B-C) Heat-maps of cell proliferation (B), and cell size (C) for *A. thaliana cuc2-3* leaf. (D-G) Heat-maps of area extension (D), growth anisotropy (E), cell proliferation (F), and cell size (G) for the *A. thaliana pin1* mutant leaf. (H-J) Model of leaf growth in the absence of marginal patterning. (H) Specified growth is homogeneous and anisotropic during early development (initiation, 1-1.85 DAI), and follow indicated tissue-dependent directions thereafter (anisotropic in the midrib/petiole, isotropic in the blade). Specified growth rates depend on tissue type and differentiation. Differentiation begins when a compound diffusing from the leaf base (green curve, graph) falls below a threshold value (dotted line). Following differentiation, growth (orange curve) decreases. (I-J) Resultant shape and distribution of growth rates and anisotropy of the model (I), compared to growth anisotropy in *cuc2-3* mutant (J). Note that growth rates decrease from the leaf tip to the base, mirroring the progression of differentiation. See also Video S3. White lines in (E,J) indicate the orientation of cell growth where anisotropy is higher than 40%. Dotted lines in (C, G) indicate leaf outlines. Scale bars, 1 cm in (A), 100 μm in (B-G and I-J).

Our data suggest that differences in tissue growth during the patterning of marginal protrusions help to create distinct leaf forms. The placement and growth of marginal protrusions requires polar auxin transport, which is controlled by the auxin efflux carrier PIN-FORMED1 (PIN1, Figures 2A and 2B) and is influenced by the transcription factor CUP-SHAPED COTYLEDON2 (CUC2) (Barkoulas et al., 2008, Bilsborough et al., 2011, Hay et al., 2006, Nikovics et al., 2006, Rast-Somssich et al., 2015). To investigate how components of the auxin-PIN1-CUC2 module influence leaf growth and differentiation, we first examined the role of auxin. Activity of this hormone, as reported by the DR5 auxin activity sensor, marks outgrowing protrusions in both *A. thaliana* and *C. hirsuta* leaves (Barkoulas et al., 2008, Hay et al., 2006). Two lines of evidence indicated that auxin activity maxima influence the rate and direction of growth. First, we found that initial growth in protrusions is rapid and anisotropic (Figures 2D, 2F, S2A, S2B, S2D, and S2E). Second, these points of rapid anisotropic growth are absent in the leaves of *C. hirsuta* and *A. thaliana* plants treated with the polar auxin-transport inhibitor, 1-N-naphthylphthalamic acid (NPA) (Figures S3A–S3J), which lack auxin maxima (Hay et al., 2006, Barkoulas et al., 2008). NPA treatment generated simple leaves in both species, and a growth pattern also observed in the leaves of *pin1* and *cuc2 A. thaliana* mutants (Figures 2G–2I and S4A–S4G; Video S2), which also lack discrete marginal auxin maxima (Bilsborough et al., 2011, Hay et al., 2006). By contrast, in addition to its role in patterning auxin maxima, CUC2 appears to inhibit growth. Following the establishment of auxin maxima, CUC2 is expressed primarily in the sinuses of *A. thaliana* leaves (Nikovics et al., 2006, Bilsborough et al., 2011), where growth and proliferation are reduced (Figures 1C, 1I, 1J, S1A, and S1T), and this growth inhibition is lost in *cuc2* mutants (Figures 2G and S4B). Thus, auxin and CUC2 shape the leaf margin by locally increasing growth at protrusions and decreasing growth at their flanks, respectively.

Video S2. Growth of Wild-Type A. thaliana and C. hirsuta Leaves Treated with NPA and A. thaliana cuc2-3 and pin1 Mutants, Related to Figures 2 and S3

**Figure S5:**
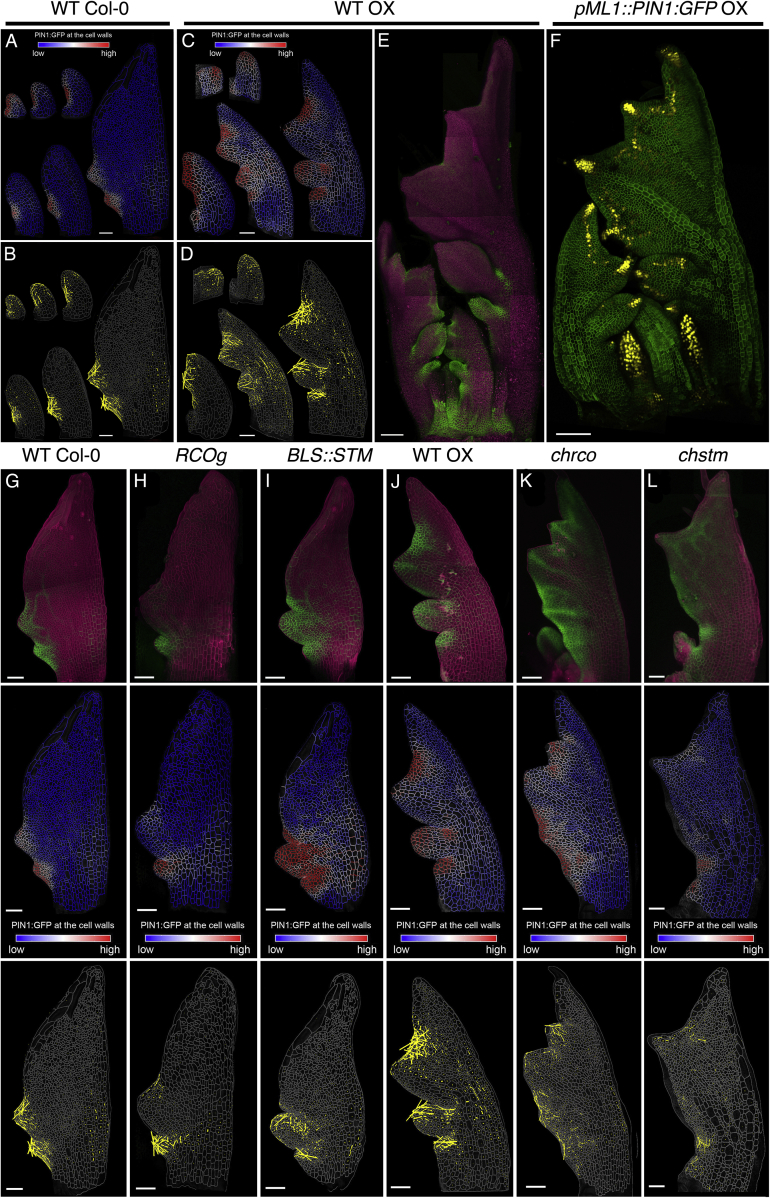
Patterns of PIN1 Expression and Polarization in Developing Leaves, Related to Figure 2, Figure 3, Figure 4 (A-B) Expression of *pAtPIN1::PIN1:GFP* in *A. thaliana* leaves. Quantification of PIN1:GFP signal at cell membranes (A) and the orientation of PIN1 polarization (B) in the epidermis. (C-E) Expression patterns of *pAtPIN1::PIN1:GFP* in *C. hirsuta* leaves. Quantification of PIN1:GFP signal at cell borders (C) and the orientation of PIN1 polarization (D) in the epidermis. (E) Confocal stacks of PIN1:GFP signal in green and autofluorescence in magenta at a later stage of development. PIN1 expression is maintained for longer in *C. hirsuta* leaves compared to *A. thaliana*. Note high PIN1 expression at early stages of leaf development and at protrusions. Color scales in (A and C) represent the intensity of GFP fluorescence. Cell outlines in (B and D) are marked in gray. Yellow lines in (B and D) indicate the orientation of PIN1 polarization in each cell (length of the lines is proportional to the strength of PIN1 polarization). Note that cells with strongly polarized PIN1 are mainly located at the tip of the early leaf primordia or at emerging protrusions. (F) The expression pattern of *pAtML1::PIN1:GFP* in *C. hirsuta* leaves with GFP signal in green and pDR5::VENUS signal in yellow. (G-L) The expression patterns of *pAtPIN1::PIN1:GFP* (top row), quantification of PIN1:GFP expression at cell borders (middle row) and the orientation of PIN1 polarization in the epidermis (bottom row) of *A. thaliana* WT (G), *RCOg* (H), and *BLS::STM* (I) as compared to *C. hirsuta* WT (J), *rco* (K) and *stm* (L) mutant leaves. The duration of PIN1 expression is extended in *C. hirsuta* WT leaves. Confocal stacks with PIN1:GFP signal in green and propidium iodide in magenta (top row). Color scales in the middle row represent intensity of GFP fluorescence. Cell outlines in bottom row are marked in gray, with yellow lines indicating the orientation of PIN1 polarization in each cell (length of the lines is proportional to the strength of PIN1 polarization). Scale bars 50 μm in A-D and G-L, and 100 μm in E-F.

To assess the role of PIN1 in tissue growth polarity in leaves (Bilsborough et al., 2011, Hay et al., 2006), we monitored its distribution during leaf development in both species. During leaf initiation, PIN1 was uniformly expressed, with its expression later coinciding with regions of active marginal patterning (Figures 2A–2B and S5A–S5E). PIN1 polarities pointed toward protrusion tips, mirroring the directions of growth at emerging protrusions (Figures 2C–2F, S5B, and S5D). We did not detect PIN1 expression in the remainder of the blade, where we observed isotropic growth in time-lapse samples (Figures 1E and 1F). In *C. hirsuta* primordia, prolonged PIN1 expression coincided with delayed differentiation (Figures 2B and S5C) and with the late emergence of protrusions in the terminal leaflet (Figure S1K). To test whether PIN1 expression can influence growth direction following differentiation, we expressed PIN1 throughout the L1 layer in *C. hirsuta*, including in the distal leaf blade where it is typically not expressed (Figure S5F). This had no apparent effect on patterning or leaf shape, consistent with previous results in *A. thaliana* (Bilsborough et al., 2011). Thus, our results indicate that PIN1 localization influences growth polarities but that its action is local rather than global and lost following differentiation.

We next assessed how marginal patterning relates to differentiation. Differentiation fronts were initiated at marginal protrusions (Figures 1C–1D, S1A–S1D, and S2A–S2F) and their formation depended on the auxin-PIN1-CUC2 module (Figures 2G–2I, S3A–S3J, and S4B–S4G). We therefore reasoned that auxin maxima may direct the formation of these differentiation fronts. To test this, we treated *A. thaliana* leaves with auxin and observed accelerated growth and differentiation (Figures 2J–2K and S3K–S3S), indicating that auxin can promote both growth and differentiation and that auxin maxima help to establish multiple differentiation fronts.

### A Computational Model of Marginal Patterning and Leaf Growth

**Figure 3:**
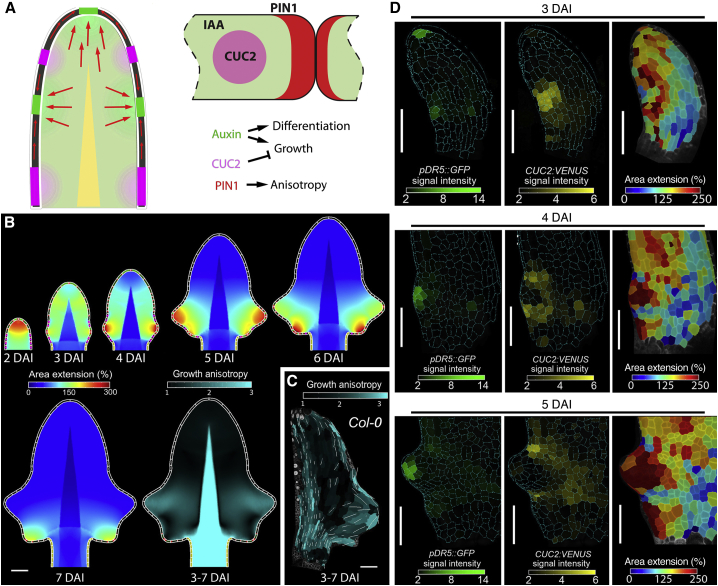
Model of Marginal Patterning and Leaf Growth (A–C) Model of *A. thaliana* wild-type leaf. (A) Margin cells at the leaf edge simulate a feedback between auxin (IAA), CUC2 and PIN1. PIN1-dependent auxin maxima in the margin promote anisotropic growth, while CUC2 inhibits growth to influence nearby blade growth. (B) Resulting leaf shape, area extension, and anisotropy distribution from the model (C) as compared to growth anisotropy in an *A. thaliana* leaf. (D) Heat-maps of *pDR5::GFP* (left panel) and *pCUC2::CUC2:VENUS* (middle panel) signal intensity, and area extension (right panel) in 3–5 DAI *A. thaliana* leaf epidermis. The time intervals for area extension heat-maps are: 3–4 DAI (3 DAI), 4–5 DAI (4 DAI and 5 DAI). White lines in (C) indicate cell growth orientation when anisotropy > 40%. Scale bars, 100 μm (B and C), 50 μm (D). See also Figure S2, Figure S4, Figure S5 and Video S3.

We developed a physically based computational model using the finite element method (FEM) to further investigate how the auxin-PIN1-CUC2 module controls the dynamic marginal growth patterns that underlie leaf shape and to understand how margin growth is integrated with overall leaf blade growth (Figure 3). We first modeled leaf growth when margin patterning was disrupted. Our results indicate that, in this case, leaf development converges on a common growth pattern characterizing *pin1* and *cuc2 A. thaliana* mutants (Figures 2G–2I and S4B–S4G) and development of both species following NPA treatment (Figures S3A–S3J). Each of these perturbations generates a simple leaf with smooth margins. We thus used this common growth pattern to characterize the bulk growth of the leaf (Figures S4H and S4I and Methods S1). We assumed that growth of the leaf depends on tissue type (blade versus midrib/petiole) and growth rates decrease with the basipetal progression of differentiation. This model reproduced the convergent development and form observed when margin patterning is perturbed, including the emergence of a radiating pattern of anisotropy at the blade-petiole junction (Figure S4I and S4J).

We then extended this model to incorporate both molecular regulation at the leaf margin (Figure 3A and Methods S1) (Bilsborough et al., 2011) and bulk interior growth. For margin growth regulation, we assumed that (1) PIN1 locally regulates growth polarities by organizing auxin maxima; (2) auxin locally increases growth rates, accelerates differentiation, and negatively regulates CUC2; and (3) CUC2 locally inhibits growth and permits the organization of auxin maxima by PIN1. The resulting model reproduces the growth patterns and leaf shapes observed during *A. thaliana* development (Figures 3B and 3C; Video S3). Thus, by combining time-lapse imaging with simulations of growth and patterning in a physically connected leaf blade, we obtained an integrated model of auxin-PIN1-CUC2-mediated growth regulation that conceptualizes how marginal and non-marginal tissue growth may interact to create a simple serrated leaf form.

Video S3. Physically Based Simulations of Leaf Development, Related to Figures 3 and S4 The model of the default growth pattern observed when marginal patterning at the leaf margin is absent, followed by the model of wild type *A. thaliana* leaf development.

### Time-Lapse Imaging of Marginal Patterning Tests Computational Model

In our simulation, we observed the emergence of an interspersed distribution of auxin activity maxima and CUC2, as has been observed in confocal micrograph snapshots (Bilsborough et al., 2011, Maugarny-Calès et al., 2019). However, it is possible that the mechanism generating this interspersed distribution cannot be inferred from snapshots. For example, an oscillatory mechanism (as in somitogenesis; Cotterell et al., 2015) could sequentially establish auxin maxima and CUC2 domains. Or distal CUC2 expression could organize the neighboring auxin maximum (as in ectopic abaxial outgrowths of *kanadi1;2* mutants; Abley et al., 2016). Both mechanisms could generate auxin maxima together with a strong CUC2 expression domain on the proximal or distal side of a protrusion. If CUC2 inhibits growth, then the asymmetric distribution of CUC2 should cause the protrusion to be asymmetric from emergence. By contrast, if a protrusion is flanked on both sides by CUC2, our model predicts it to be symmetric at initiation and to only later become asymmetric due to the proximodistal distribution of growth rates.

To investigate these possibilities, we live-imaged CUC2 and DR5 expression during serration initiation in *A. thaliana* leaves. Consistent with our model’s predictions, we observed that the auxin maximum that patterns a serration is organized in a CUC2-expression domain, such that DR5 expression precedes the elimination of CUC2 at this site (Figure 3D). As in our model, we found that high growth at the auxin maxima, juxtaposed against slow growth in adjacent CUC2-expressing sinuses, created a growth differential that leads to the emergence of a symmetric protrusion. Thus, our current model captures how local growth regulation by marginal patterning involving auxin, PIN1 and CUC2 influences leaf shape in *A. thaliana*. It also links gene activities, as studied in real time, to specific aspects of growth amount and direction.

### *In Silico* Exploration of Quantitative Differences in Development

**Figure 4:**
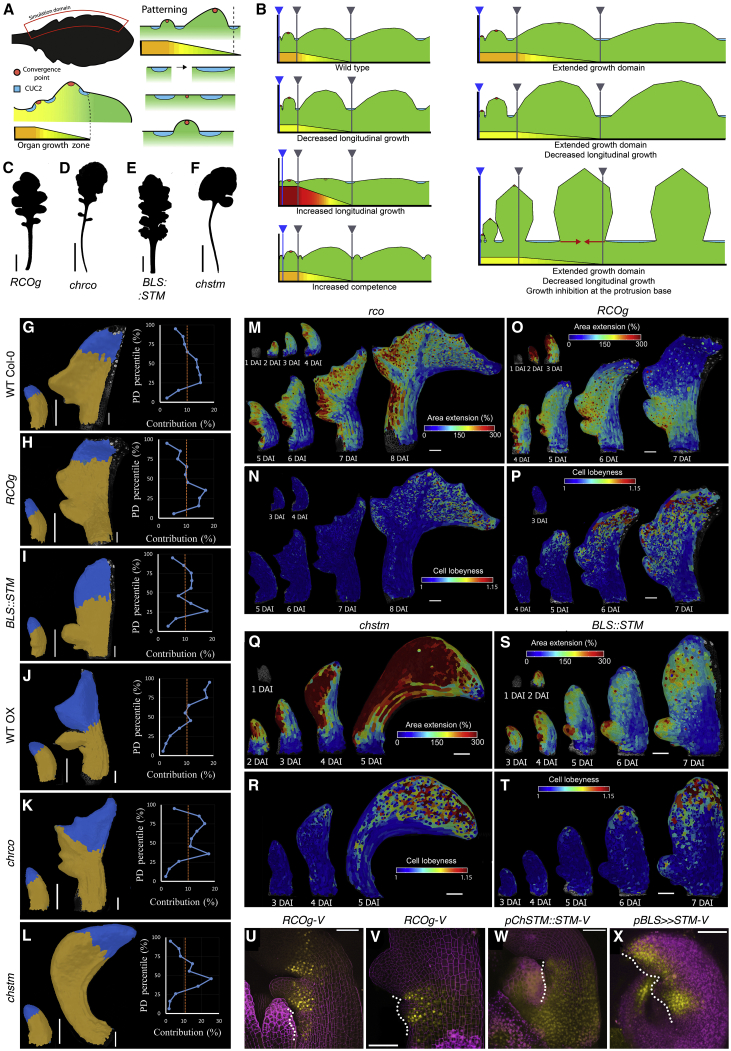
RCO and STM Together Shape Leaf Growth (A and B) Geometric model of protrusion development. (A) Principle of the simulation. Red inset indicates simulated margin area. Blade extension drives patterning. *CUC2* intervals (blue) that exceed a threshold length are broken by a convergence point (red). Distal portions of the margin differentiate at the black dotted line. Growth has a basipetal gradient (red-green scale, with red denoting regions of highest growth). (B) Effects of varying simulation parameters on protrusion form, with WT simulation acting as reference. Loss of competence to produce protrusions (blue bars, arrowheads), start and end of decreasing growth zone (gray bars, arrowheads), and growth restriction at the protrusion base (red arrows) are indicated. (C–F) Leaf silhouettes of *A. thaliana RCOg* (C) and *BLS::STM* (E) transgenic lines; *C. hirsuta rco* (D), and *stm* (F) mutants. (G–L) Left panels, lineage tracing of distal quartile (blue) of early primordia (3–7 DAI, G–K; or 2 DAI until end of the time-lapse, L), for *A. thaliana* WT (G), *RCOg* (H), *BLS::STM* (I), and *C. hirsuta* WT (J, OX), *rco* (K), and *stm* (L). Right panels, growth alignment graphs of estimated cellular contribution at final time-point (%, x axis) of cells along PD-axis at 3 DAI (y axis, 10 bins, each containing 10% of cells at 3 DAI). (M–T) Heat-maps of area extension (M, O, Q, and S) and cell lobeyness (N, P, R, and T) for *C. hirsuta rco* (M and N), and *stm* (Q and R) mutant leaves and *A. thaliana RCOg* (O and P) and *BLS::STM* (S and T) leaves. (U–X) Localization of *RCO:VENUS* (yellow) in *C. hirsuta* (U) and *A. thaliana* (V), *pChSTM::STM:VENUS* in *C. hirsuta* (W) and *BLS≫STM:VENUS* in *A. thaliana* (X) leaves; dotted-lines mark the leaf margin. Scale bars, 1 cm (C–F), and 100 μm (G–X). See also Figure S2, Figure S5, Figure S6 and Videos S4 and S5.

We next set out to explore how both global and local growth inputs are integrated to generate leaflets in *C. hirsuta* (Figure 1). To do so, we constructed a geometric model of margin development (Figure 4A) (see Methods S1; Runions et al., 2017) to examine how growth activators, repressors, and differentiation interact to form marginal protrusions. In this model, the local growth of lateral protrusions in the context of global leaf blade extension generates periodic outgrowths, with forms dependent on the model’s parameter values (Figure 4B).

We explored the parameters that affect growth at the protrusion base to evaluate their impact on margin form (Figure 4B and Video S4). In the model, decreased longitudinal growth produced more focused outgrowths. By contrast, increased longitudinal growth impeded the formation of focused protrusions by smoothing outgrowths. Increasing the duration of marginal patterning led to the initiation of intercalary protrusions but did not affect protrusion form. Extending the growth domain increased protrusion size without otherwise affecting their form. Protrusion size was further increased by both slowing longitudinal growth and by extending the growth domain. These effects coincide with the suggested role of the *KNOX* gene *STM* in retarding growth and differentiation in *C. hirsuta* leaves (Hay et al., 2006). However, protrusions still lack the narrow base found in leaflets. By introducing a domain at the protrusion base that resists longitudinal extension, the model generates outgrowths with a narrow base (Figure 4B and Video S4), thereby eliminating the characteristic asymmetric shape of serrations in *A. thaliana*. Local growth inhibition in this region is consistent with the proposed function of the *RCO* gene at the base of leaflets in *C. hirsuta* (Vlad et al., 2014). In summary, our model predicts three parameters, which together contribute to the contrasting leaf morphologies of *C. hirsuta* and *A. thaliana:* (1) a global decrease in longitudinal growth, (2) an extended duration of growth, and (3) growth inhibition at leaflet bases. We hypothesized that these parameters can be mapped to the actions of *STM* and *RCO*, with *STM* acting to globally reduce growth and delay differentiation (1 and 2) and *RCO* acting to locally inhibit growth at the base of emerging protrusions (3).

Video S4. The Geometric Model of Protrusion Development, Related to Figure 4 A wild-type simulation is shown followed by simulations where parameters affecting protrusion form are varied, including: the rate of longitudinal growth, the region of competence for protrusion generation or the length of the growth domain.

### RCO Acts Locally, and STM Acts Broadly, to Increase Leaf Complexity

**Figure S6:**
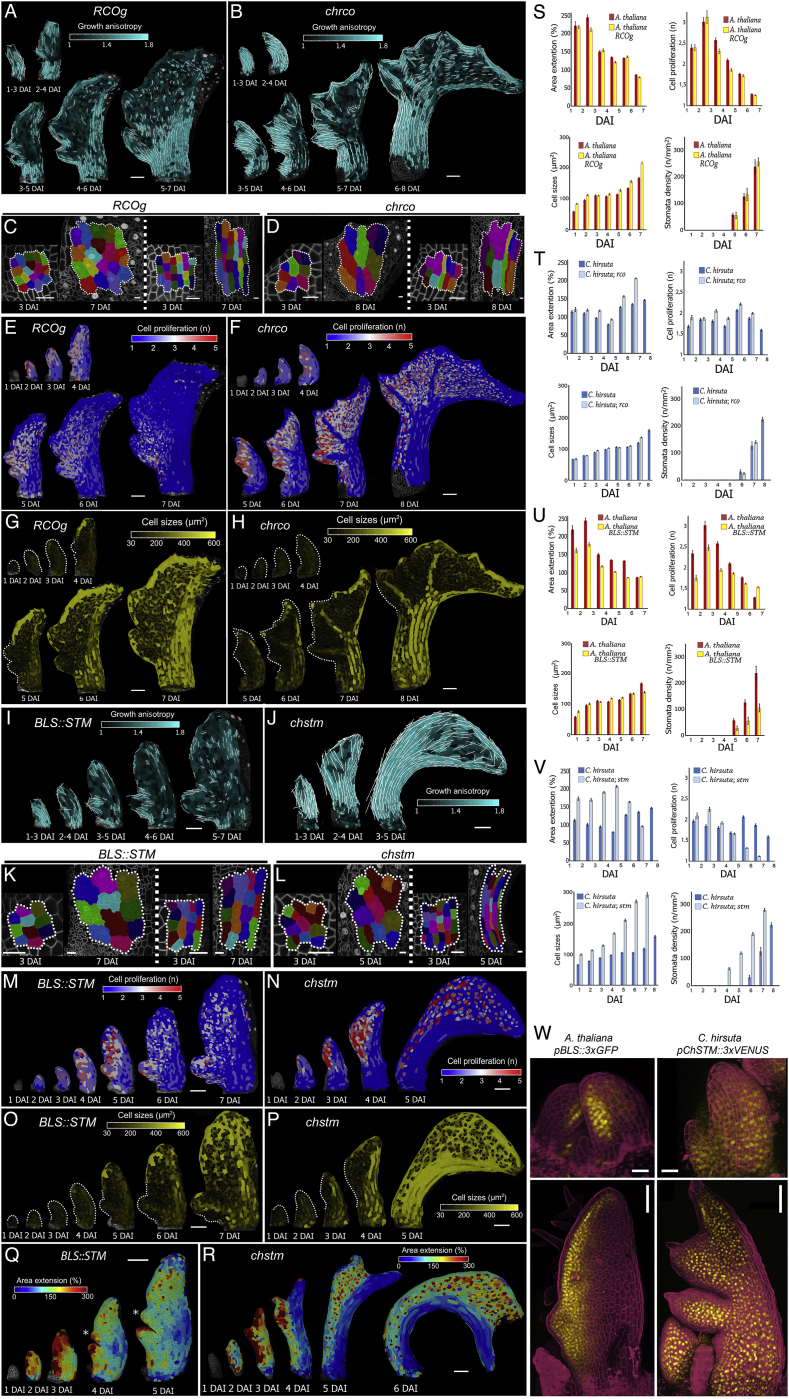
The Influence of *RCO* and *STM* on Leaf Growth, Related to Figure 4 (A-B) Heat-maps of growth anisotropy for *A. thaliana RCOg* (A), and *C. hirsuta rco* (B) leaves (white lines indicate the orientation of cell growth where anisotropy is higher than 40%). (C-D) Cell lineage tracing analysis in the leaf blade (left panels) or petiole/midrib (right panels) for *A. thaliana RCOg* (C) and *C. hirsuta rco* (D) leaves from 3-7 DAI. Colors show the correspondence between cells at 3 DAI and their clonal sectors at 7 DAI. (E-F) Heat-maps of cell proliferation for *A. thaliana RCOg* (E) and *C. hirsuta rco* (F) leaves. (G-H) Heat-maps of cell size for *A. thaliana RCOg* (G) and *C. hirsuta rco* (H) leaves. (I-J) Heat-maps of growth anisotropy for *A. thaliana BLS::STM* (I) and *C. hirsuta stm* (J) leaves (white lines indicate the orientation of cell growth where anisotropy is higher than 40%). As the *BLS::STM* sample used for 3 DAI in (M) and (N) was not captured at 1 DAI; the heat-map of anisotropy over 1-3 DAI in (I) was obtained from an independent time-lapse series. (K-L) Cell lineage tracing analysis in the leaf blade (left panel) or petiole/midrib (right panel) for *A. thaliana BLS::STM* (K) and *C. hirsuta stm* (L) leaves from 3-7 DAI. Colors show the correspondence between cells at 3 DAI and their clonal sectors at 7 DAI. (M-N) Heat-maps of cell proliferation for *A. thaliana BLS::STM* (M) and *C. hirsuta stm* (N) leaves. (O-P) Heat-maps of cell size for *A. thaliana BLS::STM* (O) and *C. hirsuta stm* (P) leaves. (Q-R) Heat-maps of area extension in an *A. thaliana BLS::STM* transgenic leaf (Q) and a *C. hirsuta stm* mutant (R). Asterisk indicates additional protrusion emerging from the leaf margin in (Q). (S-V) Quantifications of mean area extension, cell proliferation, cell size, and stomata density for *A. thaliana RCOg* (S), *C. hirsuta rco* (T), *A. thaliana BLS::STM* (U) and *C. hirsuta stm* (V) compared to WT leaves (error bars indicate SEM). (S) *A. thaliana RCOg*, n = 2-4 (stomata density) and n > 150 (remaining plots, 4 independent time-lapse series). (T) *C. hirsuta rco*, n = 3 (stomata density) and n > 80 (remaining plots, 3 independent time-lapse series). (U) *A. thaliana BLS::STM*, n = 3-4 (stomata density) and n > 100 (remaining plots, 4 independent time-lapse series). (V) *C. hirsuta stm*, n = 2-4 (stomata density) and n > 300 (remaining plots, 4 independent time-lapse series). (W) *pAtBLS::3xGFP* expression pattern in *A. thaliana* (left column) and *pChSTM::3xVENUS* expression pattern in *C. hirsuta* (right column). Confocal images with GFP or VENUS signal in yellow and propidium iodide staining in magenta. Note that tissue-dependent growth patterns are still observed in leaf primordia of *A. thaliana RCOg* (C) and *BLS::STM* (K) plants, as well as *C. hirsuta rco* (D) and *stm* (L) mutants. DAI indicates days after primordia initiation. Scale bars, 100 μm in (A-B, E-J, M-R, and W bottom panels), 20 μm in (C-D, K-L, and W top panels).

To test predictions from our geometric model in the context of mechanically connected tissues, we analyzed the roles of *RCO* and *STM* genes in leaf development. These genes are active in leaf primordia of *C. hirsuta,* but not *A. thaliana*. *STM* is expressed in the pluripotent shoot apical meristem of both species and excluded from *A. thaliana* leaves, while *RCO* is absent from the *A. thaliana* genome (Long et al., 1996, Vlad et al., 2014). To assess how these genes influence global differences in primordium growth, we compared fate maps of wild-type leaf cells at 3 DAI to those of plants with modified *STM* and *RCO* expression (Figures 4C–4L). *C. hirsuta* leaves can be distinguished from *A. thaliana*’s by the increased contribution of distal cells to the leaf blade (Figures 4G and 4J). This feature is not accounted for by *RCO*, as expressing *RCO* in *A. thaliana* leaves from its own regulatory sequence—or eliminating it in the *C. hirsuta rco* mutant—did not alter this pattern of distal cell contribution (Figures 4H and 4K). However, this feature is lost in the simple leaves of *C. hirsuta* loss-of-function *stm* mutants (Figure 4L) and appears in *A. thaliana* when *STM* expression is driven from a leaf margin promoter that is active from 2 DAI onward (Figures 4I and 4W–4X; *BLS*, Figure S6W; Shani et al., 2009). These results show that *RCO* and *STM* expression during leaf development increases leaf complexity (Figures 4C–4F) (Vlad et al., 2014, Shani et al., 2009) but that they differ in their effects on the contribution of cells to the primordium.

We then examined how the action of each gene translated into cellular behaviors. As *RCO* does not affect the contribution of distal cells to the primordium, we reasoned that it might affect leaf development locally, consistent with its proposed role as a local growth inhibitor (Vlad et al., 2014). Loss of *RCO* activity in *C. hirsuta* and introduction of *RCO* activity in *A. thaliana* did not affect the global distribution of growth (Figure 4M, 4O, and S6A–S6D and Video S5) and differentiation (Figures 4N, 4P, S6E–S6H, S6S, and S6T; for wild type see: Figures 1C and 1G, *A. thaliana*; Figure 1D and 1H, *C. hirsuta*), or marginal patterning (PIN1 expression and the order of protrusion initiation, compare Figure S5G and S5J to S5H and S5K; Figure 1C to Figure 4O; and Figure 1D to Figure 4M). Instead, when expressed in either species, RCO inhibited local growth (Figure 4M, 4O, and S2G compared to Figures 1C and 1D; S2A) within its restricted expression domain at the base of emerging protrusions (Figures 4U and 4V). Thus, *RCO* is required for local growth repression at the base of protrusions but has no apparent global effects on leaf development.

Video S5. Growth of A. thaliana RCOg and C. hirsuta rco mutant Leaves, Followed by Growth of A. thaliana BLS::STM and C. hirsuta stm Leaves, Related to Figure 4

Our fate maps show that *STM* increases the relative contribution of distal cells to the primordium (Figures 4G–4L). We thus reasoned that it might broadly influence leaf development. Time-lapse imaging of *C. hirsuta stm* mutants and *A. thaliana BLS::STM* leaves confirmed that STM broadly affects patterns of growth, proliferation, and differentiation. Growth, proliferation, and differentiation were strongly accelerated in *C. hirsuta stm* mutant leaves (Figures 4Q, 4R, S6J, S6N, S6P, S6R, and S6V; Video S5), concomitant with an early decrease in PIN1 expression (Figures S5J and S5L) and a reduction in the lateral growth of emerging leaflets (Figure 4Q). Conversely, compared to *A. thaliana* wild-type leaf primordia (Figures 1C and S1A), *BLS::STM* primordia showed significantly reduced growth and proliferation (Figures 4S, S6I, and S6U and Video S5), delayed differentiation (Figures 4T and 1G), a broader domain of PIN1 expression and prolonged margin patterning (Figure S5G, S5I, and S6Q). Growth reduction in *BLS::STM* leaves was progressively restricted to proximal regions of the primordium (Figures 4S, S6M, and S6O) and was most apparent adjacent to emerging protrusions, where differentiation was particularly protracted (compare Figure 1G and S2C with Figures 4T and S2L). Nevertheless, increased growth at protrusion tips was comparable to WT but was maintained for longer (Figure 4S, S2J, and S2K). Thus, expressing *STM* in *A. thaliana* leaves increases the contribution of cells in distal and lateral regions to the early primordium (Figures 4G and 4I), shifting cellular growth patterns toward those of *C. hirsuta* (compare Figures 1C–1H, 4S–4T, S1A, S1B, S6I and S6M). Our observations suggest that *STM* retards early growth while delaying tissue maturation, thus prolonging the duration of growth and patterning. Notably, this STM-dependent redistribution of leaf growth might account both for the formation of lateral leaflets and the broad terminal leaflet that distinguish *C. hirsuta* from *A. thaliana*, consistent with the loss of both these features in *C. hirsuta stm* mutants (Figures 4G, 4I, 4J, and 4L).

**Figure 5:**
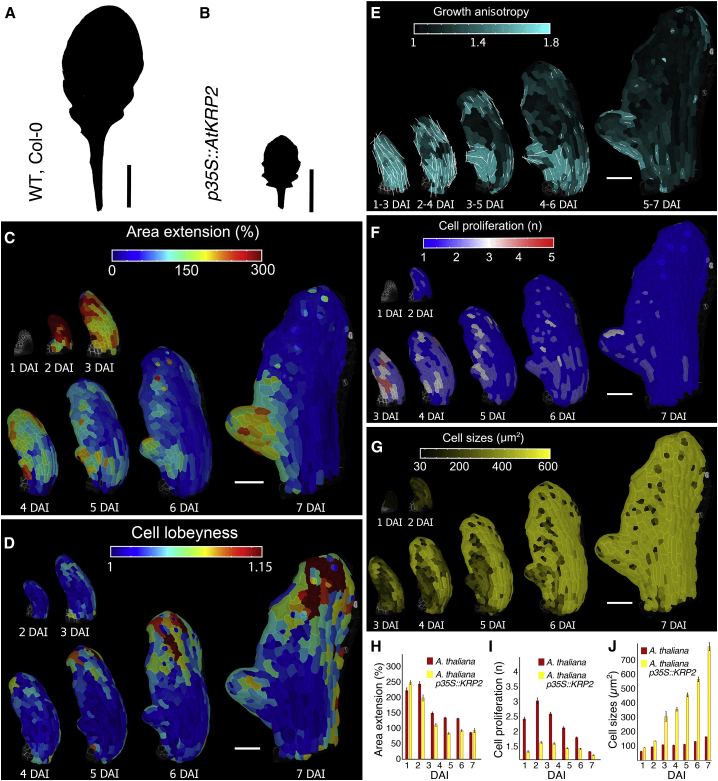
Increased Complexity of *A. thaliana p35S::KRP2* Leaves (A and B) Silhouettes of *A. thaliana* WT (A), and *p35S::KRP2* transgenic line (B) leaves. (C–G) Heat-map of area extension (C), cell lobeyness (D), growth anisotropy (E), cell proliferation (F), and cell size (G) for the *A. thaliana p35S::KRP2* leaf. (H–J) Quantifications of mean area extension (H), cell proliferation (I), and cell size (J) for *A. thaliana p35S::KRP2* leaves compared to WT leaves (error bars indicate SEM). *A. thaliana p35S::KRP2*, n = 135-979 (H, 5 independent time-lapse series), n = 123–1,225 (I, 5 independent time-lapse series), n = 135–1,322 (J, 5 independent time-lapse series). White lines in (E) indicate cell growth orientation where anisotropy is higher than 40%. Scale bars, 1 cm (A and B), and 100 μm (C–G)

We observe that *STM* increases leaf complexity by delaying differentiation. However, accelerated differentiation can also increase leaf complexity, as seen in *A. thaliana* plants overexpressing *KIP-RELATED PROTEIN2* (*KRP2*) under the *35S* promoter (Figures 5A and 5B; De Veylder et al., 2001). Thus, both delayed and accelerated differentiation can increase leaf complexity. We investigated this contradiction by recording time-lapse images of *p35S::KRP2* leaves (Figure 5C–5J), which confirmed the early onset of differentiation, relative to wild-type (∼3 DAI versus ∼5 DAI in wild type; compare Figures 5C–5J with Figures 1C, 1G, S1A, and S1C). *p35S::KRP2* leaves initiated slow-growing symmetric serrations and sharp sinuses that were maintained over time, unlike in wild-type leaves (Figure 5C). Thus, a common feature of *BLS::STM, RCOg* and *p35S::KRP2* leaves was decreased growth in the regions surrounding initiating protrusions. These observations indicate that a slow growth context enables the maintenance of more prominent protrusions, regardless of the differentiation status of surrounding tissue, as also suggested by our geometric model (Figure 4B).

### Modulating Both Local and Global Growth Produces a Dissected Leaf

**Figure 6:**
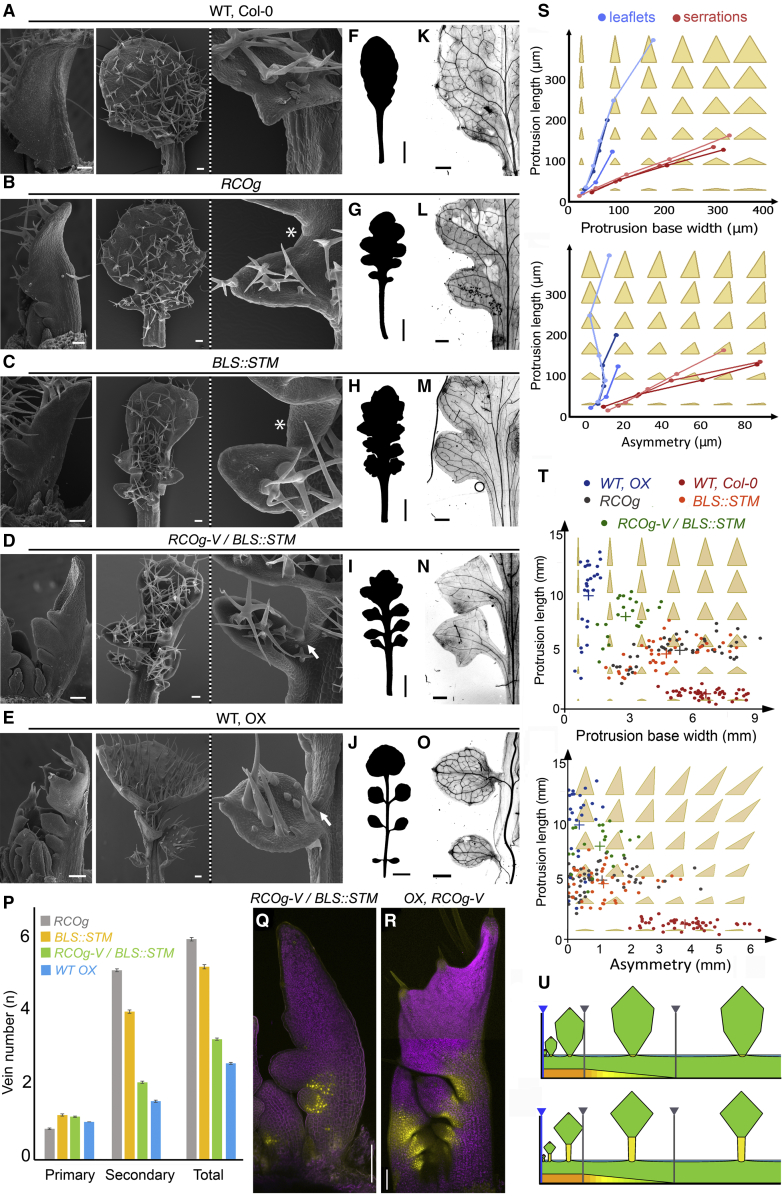
Reconstructing Dissected Leaf Shape by Combining *RCO* and *STM* in *A. thaliana* Leaves (A–E) Scanning electron micrographs of *A. thaliana* WT (A), *RCOg* (B), *BLS::STM* (C), *RCOg-V; BLS::STM* (D), and *C. hirsuta* WT (OX) (E) leaves. Insets: close-up views of marginal protrusions. Leaf blade between protrusions (white stars) and absence of blade between leaflets (white arrow) are shown. (F–J) Silhouettes of fully developed eighth rosette leaves of *A. thaliana* WT (F), *RCOg* (G), *BLS::STM* (H), *RCOg-V*; *BLS::STM* (I), and *C. hirsuta* WT (J). (K–P) Vascular architecture in protrusions of *A. thaliana* WT (K), *RCOg* (L), *BLS::STM* (M), *RCOg-V; BLS::STM* (N), *C. hirsuta* WT (O) leaves, and (P) vein number at protrusion bases (n = 21). Error bars indicate SE. (Q and R) Expression of *pRCO::RCO:VENUS* fusion protein (yellow) in *RCOg-V; BLS::STM A. thaliana* (Q) and *C. hirsuta* (R) leaves. (S and T) Analysis of protrusion geometry in time-lapse series (S) and mature leaves (T) of *C. hirsuta* (blue), and *A. thaliana* (red), *RCOg-V* (gray), *BLS::STM* (orange), and *RCOg; BLS::STM* (green) for protrusion length versus width (top), and protrusion length versus asymmetry (bottom); crosses indicate the mean for each background. Triangle shape captures the base to protrusion width (distance between sinuses), the height to protrusion length, and asymmetry to the length difference between left and right triangle edges. (U) Geometric model extension to account for leaflets. Yellow at leaflet base indicates zone of RCO action. (Top) Strong RCO repression at leaflet rachis junction yields sessile leaflets. (Bottom) Extending zone of RCO action into the leaflet base produces petiolate leaflets. Scale bars, 100 μm in (A-E and Q-R), 1 cm in (F-J), and 1 mm in (K–O). See also Video S6.

Our observations indicate that differences in *A. thaliana* and *C. hirsuta* leaf development include the local modification of growth during patterning and a global redistribution of growth that results from delayed differentiation (Figures 1, 2, and 3). Alone, neither of these features generated a dissected leaf (Figures 4 and 5), leading us to hypothesize that they act together to produce leaflets. In agreement with this hypothesis, the combined expression of *RCO* and *STM* in *A. thaliana* double transgenic plants reproduced key aspects of the *C. hirsuta* dissected leaf form (Figures 6A–6J), including a broad terminal leaflet. In *A. thaliana* plants expressing *RCOg-VENUS* (*RCOg-V*) and *BLS::STM* transgenes, protrusions were completely separated from each other and supported by a narrow base (Figure 6I). *RCO* expression localized to the petiolules of both terminal and lateral leaflets (Figures 6Q and 6R), and the vascular architecture of *RCOg-V*; *BLS::STM* leaves resembled that of *C. hirsuta* rather than *A. thaliana* leaves (Figures 6K–6P).

These results suggest that growth inhibition at the base of emerging protrusions, combined with an increase in their outgrowth, can convert a serration into a leaflet. To test this idea, we quantified the overall form of emerging protrusions in our time-lapse series by using a triangle to approximate protrusion shape (Figure 6S, Methods S1). Our analysis showed that during serration development, the protrusion base quickly increased in width and gradually increased in length. By contrast, in leaflets, the protrusion base remained narrow while the protrusion elongated substantially. Notably, leaflets remained symmetric, whereas serrations adopted an asymmetric form during development (Figure 6S). Measurements of mature leaves are consistent (Figure 6T) and, thus, indicate that leaflets result from the combined effects of *BLS::STM* and *RCOg* on shape. Qualitatively, the changes in serration and leaflet shapes (Figures 6S and 6T) were consistent with the changes in growth predicted by the geometric model to increase the prominence and symmetry of protrusions (Figure 4B), specifically: the local effects of *RCO* inhibiting growth at the protrusion base, and the global effects of *STM* in slowing the rate of growth and differentiation, while prolonging growth duration. However, the simulations shown in Figure 4B evidently do not reproduce the narrow stalk observed in leaflets of *A. thaliana RCOg-V*; *BLS::STM* (Figure 6I and 6N) and *C. hirsuta* (Figure 6J and 6O).

To conceptualize the combined effects of *RCO* and *STM* on leaf form, we thus returned to the geometric model of *A. thaliana* margins. Inspired by our biological data (Figure 6I, 6N, and 6Q), we explored the effects of manipulating the amount and domain of growth repression at protrusion bases (Figure 6U and Video S6). Increasing growth inhibition at the protrusion base transformed lobes into leaflet shapes but did not produce a stalked base. Extending the inhibition zone at the protrusion base to match *RCO* expression (Figures 6Q and 6R) yielded a leaflet supported by a narrow stalk. Taken together, our data clarify how the combined action of RCO and STM during leaf development is sufficient to account for key aspects of simple versus dissected leaf development despite the many multi-scale interactions that influence leaf shape. Both genes contribute to slow growth at the protrusion base, which enables the lateral growth anisotropy of protrusions to generate discrete leaflets (Figures 1C–1F and 4). However, STM also contributes to the sustained longitudinal extension that allows leaflets to separate. Thus, growth inhibition both shapes the outgrowths driven by anisotropic lateral growth (2–6 DAI) and enables the anisotropic extension of the central leaf stalk (termed rachis) to separate leaflets (7–8 DAI) (Figures 1D and 1F). The relatively mild phenotypes that distinguish *A. thaliana RCOg-V*; *BLS::STM* plants from *C. hirsuta* might reflect other differences in gene expression between the leaf primordia of the two species, including differential expression of several meristem transcription factors (Gan et al., 2016).

Video S6. A Geometric Model of Protrusion Development at the Leaf Margin with Extended Growth Domain, Decreased Longitudinal Growth, and Strong Growth Repression at the Base of Protrusions, Related to Figure 6 The lateral extent of growth repression in the protrusion base is varied between simulations.

## Discussion

**Figure 7:**
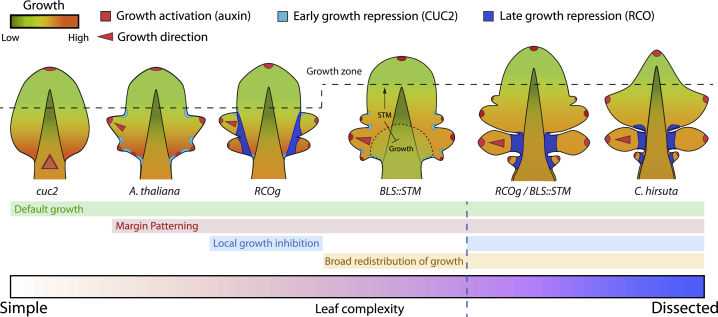
How Genetic Factors Influence Growth to Shape Divergent Leaf Forms Schematic representation of *A. thaliana* and *C. hirsuta* leaf shapes; green-orange gradients represent growth rates, dashed line represents distal boundary of growth zone. Without auxin-based patterning, species have a common growth pattern, characterized by a basal growth zone and tissue-dependent growth polarities (midrib/petiole, anisotropic; blade, isotropic), producing simple leaves with a smooth margin (as seen in *A. thaliana cuc2-3* mutants, where it may represent a default growth pattern). Auxin, PIN1 and CUC2 pattern alternate regions of growth repression and anisotropic growth activation along the leaf margin. Margin patterning creates outgrowths (serrations in *A. thaliana*). Local growth inhibition by RCO accentuates the growth differences created by margin patterning, generating more pronounced outgrowths (as in *A. thaliana RCOg*). STM functions to broadly redistribute growth by extending the growth zone, which increases leaf complexity. STM slows differentiation and growth in median and proximal regions, and prolongs growth and patterning in distal and lateral parts of leaf primordia (as in *A. thaliana BLS::STM*). This has two consequences: (1) it provides a second mode of growth repression, which creates leaflets when RCO is present. (2) It increases the relative contribution of lateral and distal regions to final leaf form, creating a broader leaf tip. Together, RCO and KNOX actions account for key differences in growth between *C. hirsuta* and *A. thaliana* leaves, as demonstrated by reconstructing a dissected leaf form in *A. thaliana RCOg-V; BLS::STM*.

We investigated how key regulators of organogenesis influence the rate and orientation of growth, cell proliferation, and the timing of cellular differentiation to generate different leaf forms. To do so, we acquired real-time, cellular-level growth data at an unprecedented resolution. This information, coupled with theoretical analysis, guided the *in vivo* reconstruction of a complex morphological trait from its cell-level constituent elements (Figure 7).

At the single-gene level, we resolved how *CUC2* can both stimulate and repress growth (Bilsborough et al., 2011, Blein et al., 2008, Maugarny-Calès et al., 2019); first, it triggers auxin activity maxima within its own domain; then, it represses cell growth as a separate effect in the region flanking these maxima (Figure 3). We also show how the *KNOX* gene, *STM*, influences leaf form by slowing growth and delaying differentiation in the proximal domain where marginal patterning occurs. These effects enable the auxin-PIN1-CUC2 patterning module to increase both the number and prominence of marginal protrusions. These interactions also concur with previous findings on how *KNOX* genes act, including their possible repression by auxin (Barkoulas et al., 2008, Blein et al., 2008, Bolduc et al., 2012, Hay et al., 2006, Heisler et al., 2005, Richardson et al., 2016). By explicitly considering dynamic cell-level growth, we extend our understanding of how these molecular regulators create simple versus complex leaves beyond the view that they mainly influence local tissue identities in the leaf (Bar and Ori, 2014, Hay and Tsiantis, 2010). A future challenge is to resolve how other regulators affect cell growth, such as *NGATHA* and *CINCINNATA* genes, which appear to prevent marginal outgrowth independently of *CUC2* (Alvarez et al., 2016).

At the organ level, we show how local foci of genetically controlled growth anisotropy at the leaf margin interact with a broadly isotropic, organ-wide growth pattern to produce leaf form. In this context, the generation of leaflets versus serrations involves both the global modification of growth by STM and local growth inhibition conferred by RCO. These effects accentuate the non-uniform growth created by the patterning of leaf margins, thus shaping form (Figure 7). Notably, the growth repressive effects of RCO do not propagate broadly, despite leaf cells being mechanically interconnected by their walls. It will be interesting to explore the biophysical basis of this restricted propagation of RCO-dependent growth inhibition, which may also limit the potentially pleiotropic effects of evolutionary changes to *RCO* expression (Vlad et al., 2014).

Finally, our work sheds new light on the regulatory logic that connects cell- and tissue-scale effects of molecular regulators. Specifically, we found that auxin activity maxima increase growth while accelerating differentiation, while STM proteins have the opposite effect on both cellular growth and differentiation. Consequently, unlike growth regulators that predominantly influence the amount of growth (e.g., RCO), STM and auxin maxima strongly influence both growth amount and its duration, where duration depends on the proliferative status of cells. In this way, the opposing effects of auxin and STM are both self-limiting. Auxin activity increases growth, but its potential to alter form is limited by the induction of differentiation. STM delays differentiation, prolonging proliferation and patterning, but its potential is limited by its capacity to retard growth. The logic of these dual activities is similar to an incoherent feed-forward loop (Alon, 2006), a well-known gene-regulatory architecture in which a single, upstream regulator both inhibits and promotes the activity of a downstream target. These networks can provide temporally limited activation (i.e., a pulse of activity) in response to activation by the upstream regulator. In growing tissues, the opposing regulation of growth and differentiation affects the spatial-temporal distribution of growth by acting inversely on growth duration and amount. This regulatory logic might be well-suited for controlling the shape of determinate organs, by allowing fine-grained geometric changes to occur during development without dramatic changes to organ size.

## STAR★Methods

### Key Resources Table

**Table:** 

REAGENT or RESOURCE	SOURCE	IDENTIFIER
**Chemicals, Peptides, and Recombinant Proteins**		

Propidium iodide (PI)	Sigma-Aldrich	87-51-4
Indole-3-Acetic Acid (IAA)	Sigma-Aldrich	I0901.0100
Plant Preservative Mixture (PPM)	Plant Cell Technology	250
1-N-naphthylphthalamic acid (NPA)	Sigma-Aldrich	132-66-1
Roti Histol	Roth	6640

**Experimental Models: Organisms/Strains**		

*A. thaliana*: *pin1* (RCR-genotyped to be insertion GABI-Kat -051A10)	Kierzkowski et al., 2013	Cris Kuhlemeier, UniBe
*A. thaliana*: *cuc2-3*	Nikovics et al., 2006	Patrick Laufs, Institut Jean-Pierre Bourgin
*A. thaliana*: *pUBQ10::acyl:TDT*	Segonzac et al., 2012	Elliot Meyerowitz, Caltech
*A. thaliana*: *pUBQ10::acyl:YFP*	Willis et al., 2016	Raymond Wightman, University of Cambridge
*A. thaliana*: *pin1 x pUBQ10::acyl:YFP*	This study	N/A
*A. thaliana*: *cuc2-3 x pUBQ10::acyl:YFP*	This study	N/A
*A. thaliana*: *pBLS::3xGFP*	This study	N/A
*A. thaliana*: *pDR5::GFP*	Friml et al., 2003	Gerd Jurgens, Max Planck Institute for Developmental Biology
*A. thaliana*: *pUBQ10::acyl:TDT x pCUC2::CUC2:VENUS x pDR5::GFP*	This study	N/A
*A. thaliana: pBLS≫STM:VENUS*	This study	N/A
*A. thaliana: pPIN1::PIN1:GFP*	Heisler et al., 2005	N/A
*A. thaliana*: *p35S::KRP2*	This study	NA
*A. thaliana: RCOg:VENUS* (translational fusion of VENUS to RCO in genomic context in pMLBART)	Vuolo et al., 2016	Miltos Tsiantis, MPIPZ
*A. thaliana*: *pBLS::STM*	Shani et al., 2009	Yuval Eshed, Weizmann Institute Naomi Ori, Hebrew University
*A. thaliana*: *pBLS::STM x pUBQ10::acyl:YFP*	This paper	N/A
*A. thaliana*: *RCOg*	Vlad et al., 2014	Miltos Tsiantis, MPIPZ
*A. thaliana*: *RCOg* x *pUBQ10::acyl:YFP*	This study	N/A
*A. thaliana*: *p35S::KRP2* x *pUBQ10::acyl:YFP*	This study	N/A
*A. thaliana: pBLS::STM x pAtPIN1::PIN1:GFP*	This study	N/A
*A. thaliana: RCOg x pPIN1::PIN1:GFP*	This study	N/A
*A. thaliana: RCOg:VENUS x pBLS::STM*	This study	N/A
*C. hirsuta*: *rco*	Vlad et al., 2014	Miltos Tsiantis, MPIPZ
*C. hirsuta*: *stm*	Rast-Somssich et al., 2015	Miltos Tsiantis, MPIPZ
*C. hirsuta*: *stm x pUBQ10::acyl:YFP*	This study	N/A
*C. hirsuta*: *rco x pUBQ10::acyl:YFP*	This study	N/A
*C. hirsuta*: *pChSTM::3xNLS-VENUS*	This study	N/A
*C. hirsuta*: *pUBQ10::acyl:YFP*	This study	N/A
*C. hirsuta*: *pPIN1::PIN1:GFP*	Barkoulas et al., 2008	Miltos Tsiantis, MPIPZ
*C. hirsuta*: *pML1::PIN1:GFP x DR5::VENUS*	This study	N/A
*C. hirsuta: stm x pPIN1::PIN1:GFP*	This study	N/A
*C. hirsuta: rco x pPIN1::PIN1:GFP*	This study	N/A
*C. hirsuta: pChSTM::ChSTM:VENUS*	Rast-Somssich et al., 2015	Miltos Tsiantis, MPIPZ

**Recombinant DNA**		

*pUBQ10::acyl:YFP*	Willis et al., 2016	Raymond Wightman, University of Cambridge
*pBLS::STM; pBLS::LhG4*	Shani et al., 2009	Yuval Eshed, Weizmann Institute Naomi Ori, Hebrew University
*pPIN1::PIN1:GFP*	Heisler et al., 2005	N/A
*pML1::PIN1:GFP*	Bilsborough et al., 2011	Miltos Tsiantis, MPIPZ
*pBLS::3xGFP*	This paper	N/A
*pChSTM::3xNLS-VENUS*	This paper	N/A
*OP::STM:VENUS*	This paper	N/A
*pMLBART- STM:VENUS*	Heisler et al., 2005	N/A
*pVTOp*	Hay et al., 2006 and references therein	Miltos Tsiantis, MPIPZ

**Software and Algorithms**		

Fiji	NIH	https://fiji.sc/
Physically based simulations (C++/VVe)	This paper	Available upon request
Geometric models of margin development (L+C/lpfg)	This paper	Available upon request
MorphoGraphX	Barbier de Reuille et al., 2015	http://www.mpipz.mpg.de/MorphoGraphX/
VVe	Smith et al., 2004	http://www.algorithmicbotany.org
LPFG	Karwowski and Prusinkiewicz, 2003	http://www.algorithmicbotany.org
Lineage tracing script (Python)	Barbier de Reuille et al., 2015	http://www.mpipz.mpg.de/MorphoGraphX
FantaMoprh	Abrosoft	http://www.fantamorph.com/

### Contact for Reagent and Resource Sharing

Further information and requests for resources and reagents should be directed to and will be fulfilled by the Lead Contact, Miltos Tsiantis (tsiantis@mpipz.mpg.de).

### Experimental Model and Subject Details

#### *Arabidopsis thaliana* and *Cardamine hirsuta*

All transgenic plants and mutants were generated in the Col-0 background for *A. thaliana* and in the Ox background for *C. hirsuta* (Hay et al., 2006).

#### Growth Conditions

Plants were grown on soil in a growth chamber under long day conditions (16 h of illumination, 110 μEm^-2^s^-1^) at 20 ± 2°C, with 65 ± 10% relative humidity. For time-lapse experiments, soil grown plants were transferred at 11 days after germination to 1/2 MS medium including vitamins (Duchefa Biochem, M0222.0050) supplemented with 1% sucrose, 0.1% PPM and grown in long day conditions.

### Method Details

#### Construction of transgenes

All transgenes were constructed using standard cloning techniques. All finished constructs were verified by sequencing. *BLS::3xGFP*: *BLS* promoter (7.1kb upstream of BLS) in pDRIVE vector (gift from Yuval Eshed lab). *BLS* was excised by PstI and BamHI and inserted upstream of 3xNLS-GFP in pBJ36. BLS::3xGFP was excised by NotI and inserted into pMLBART. *pChSTM::3xNLS-VENUS*:*ChSTM* promoter in *pGEM-T-EASY* as described before (Rast-Somssich et al., 2015). The *pChSTM* fragment was excised by SalI and BamHI and inserted into pBJ97 upstream of *3xNLS-VENUS*. *pChSTM::3xNLS-VENUS* was then excised by NotI and inserted into pMLBART. *STM:VENUS* in pMLBART was a gift from Markus Heisler lab (Heisler et al., 2005). To construct *OP::STM:VENUS*, *STM:VENUS* was inserted in pVTOp (Hay et al., 2006 and references therein). We used the *BLS* promoter (Shani et al., 2009) to express STM in a minimal domain that is sufficient for leaflet formation in *A. thaliana*. The genomic locus of *C. hirsuta STM* was not sufficient for this purpose because *gChSTM* expression is active but not sustained in *A. thaliana* leaves and does not recreate the endogenous pattern observed in *C. hirsuta* (Rast-Somssich et al., 2015). For the *AtML1::PIN1:GFP* construct in *C. hirsuta*, we confirmed that this construct complements when introduced in a *C. hirsuta pin1* background.

Plant transformations were performed using the floral dip method, using *Agrobacterium tumefaciens* strain GV3101. For each construct, a minimum of 10 independent lines were self-pollinated to obtain T2 seeds. Plants carrying two or more transgenes were produced by crossing and analyzed in the F2 or F3 generation.

#### Chemical treatments

For 1-N-naphthylphthalamic acid (NPA) treatment, ½ MS medium supplemented with 1% sucrose was supplemented with 10μM NPA. The same concentration was added to the water used to submerge plants during confocal observations. For auxin treatment, 1μM indole-3-acetic acid (IAA, Fluka) was used as described for NPA.

#### PI staining

Seedlings were submerged in PI solution (10 mg/mL) for 5-10 min and then imaged immediately using a Leica SP8 up-right confocal microscope.

#### Phenotypic and histological analysis

To obtain leaf silhouettes and quantify leaf margin protrusions, the 8^th^ leaf of 5-weeks-old plants was removed, flattened onto clear adhesive on paper and scanned. Protrusions were measured in Fiji and analyzed as described in Methods S1.

To visualize vascular architecture, chlorophyll was eliminated using a graded ethanol series from 50% to 100% (v/v), and subsequent clearing with 50% to 100% (v/v) Roti Histol, followed by incubation with 50% glycerol overnight. Images were taken using a Nikon SMZ 1500 microscope equipped with a Nikon DS-Fi2 camera.

#### Microscopy and image analysis

All confocal imaging was performed using a SP8 upright confocal microscope equipped with a long working-distance water immersion objective (AP 20x/0.8 or AP 40x/0.8; Leica). Excitation was performed using an argon laser with 488 nm for GFP and PI, 514 nm for VENUS, YFP and PI. Images were collected at 529-545 nm for VENUS and YFP, 499-526 nm for GFP, 600-660 nm for PI and 660-749 nm for chlorophyll auto-fluorescence. For reporter gene analysis, cell walls were visualized after staining with 0.1% propidium iodide. For scanning electron microscopy, samples were prepared as described in Bilsborough et al. (2011). Imaging was performed using a JSM-5510 microscope (Joel). Images were processed and analyzed using Photoshop (Adobe) and MorphGraphX software (Barbier de Reuille et al., 2015). Quantification of fluorescence signals for each cell in the L1 layer (1-6 μm from the surface) was performed as described previously (Barbier de Reuille et al., 2015). Signal intensity of *CUC2::CUC2:VENUS* and *pDR5::VENUS* was quantified for the entire cell. *pPIN1::PIN1:GFP* signal intensity was quantified for each cell membrane based on the 1 μm region adjacent to the cell wall. PIN1:GFP orientations were assessed for each cell using the polarization plugin in MorphoGraphX (see Methods S1).

#### Growth tracking experiments

For growth analysis, cotyledons and older leaf primordia were removed from 11-days-old soil-grown plants to expose the apex for imaging. Only plants with undamaged roots, hypocotyls and emerging leaf primordia were used for time-lapse experiments. Dissected plants were then transferred into Ø60 mm Petri dishes filled with ½ MS medium including vitamins (Duchefa Biochem, M0222.0050) supplemented with 1.5% plant agar, 1% sucrose and 0.1% plant protective medium (Plant Cell Technology). To visualize cell outlines *pUBQ10::acyl:YFP* (Willis et al., 2016) or propidium iodide was used. During each experiment, at least half of the abaxial epidermis of the leaf was imaged at 24 h intervals using a Leica SP8 confocal microscope. Adult leaves 8 ± 1 were imaged for all genotypes except for *C. hirsuta stm* mutants which produce variable and reduced leaf number (Rast-Somssich et al., 2015) and where primordia as equivalently staged as possible to wild-type were observed. Between imaging, samples were transferred to a growth chamber and cultured *in vitro* under standard long-day conditions. Confocal stacks were acquired at 512x512 resolution, with 0.5-0.8 μm distance in Z-dimension, and no averaging to minimalize imaging stress. The resulting confocal time-lapse series were then analyzed using MorphoGraphX as described in Vlad et al. (2014). For samples that were larger than the scanning area, acquired stacks were stitched using MorphoGraphX. After cells were segmented, parent relations between successive days were determined manually in MorphoGraphX. These were used to calculate cell area, area extension, cell proliferation and growth anisotropy for each cell. The extent of lobing in pavement cells (lobeyness) was calculated as in Sapala et al. (2018) using a MorphoGraphX plugin and taking the ratio of each cell’s perimeter to that of its convex-hull (the smallest convex shape containing the cell). Lineage tracing analysis was performed automatically in MorphoGraphX. To compute corresponding cell lineages over multiple observations (i.e., multiple days), a custom python script was used that linked the parent relations between successive days to provide lineage relations over larger time-periods. We used lineages computed as described above to analyze protrusion shape and compute growth alignment graphs (see Methods S1), and the blade, petiole/midrib and slow growing sinus cells adjacent to emerging protrusions in *A. thaliana* and *C. hirsuta* (Figure 1 O,P). The blade, petiole/midrib and sinus cells in 3 and 7 DAI WT leaf primordia were identified by: 1) labeling the blade and petiole/midrib regions at 7 DAI based on cell shape, growth and proliferation, 2) mapping these regions to 3 DAI using lineage tracing, 3) marking the slow growing sinuses between protrusions, and 4) mapping the marked sinuses to 7 DAI.

Heat-maps for area extension, anisotropy and proliferation between two time-points are displayed on the later time-point (e.g., Leaf area extension for 3-4 DAI is visualized on the leaf at 4 DAI), with the exception of Figure 3L. In Figure 3L, area extension is visualized on the first time-point for 3 and 4 DAI, to make relations between *pDR5::GFP* and *CUC2::Venus* signal intensity and growth more apparent. Unless otherwise indicated, the time interval visualized for area extension, anisotropy and proliferation heat-maps is 1 day. Representative time-lapse series shown in main and supplemental figures were obtained from a single time-lapse experiment, with the exception of wild-type *C. hirsuta* and *chrco* leaves, as well as *A. thaliana BLS::STM*, *35S::KRP2* and *RCOg* leaves, where the first days of observation (1-2 or 1-3 DAI) were obtained from other time-lapse series. A minimum of 3 time-lapse series were performed for each genotype. These covered a period from leaf primordium emergence until up to 7-8 days after primordium initiation (DAI).

#### Movies

Movies of time-lapse sequences (Movies S1-2, S5) were created using images visualizing the average growth of each cell and its neighbors. Images were morphed using FantaMorph software (Abrosoft).

#### Modeling

Detailed description of all models can be found in Methods S1.

### Quantification and Statistical Analysis

#### Statistical analysis

Statistical analysis was performed using Microsoft Excel. Error bars in figures represent standard errors of the mean (SEM).

#### Replication for imaging data

Replication for imaging data shown in main and supplementary figures is provided below.

**Table:** 

Replication of imaging data		
	Figure panels	Replication
***A. thaliana*: WT**		

time-lapse	Fig. 1 C, E, G, I, J; 2D; 3C; S1 A, C, E, L-O; S3 O	5
time-lapse protrusion	Fig. S2 A-C	5
Sinus initiation in the blade (time-lapse)	Fig. 1 O	5
Growth/Proliferation vs PD-position (growth alignment map)	Fig. 1 K, M	3
Protrusion growth alignment map	Fig. S1 T	3
Primordium contribution (growth alignment map)	Fig. 4 G	5
*pPIN1::PIN1:GFP* expression	Fig. 2 A, C; S5 A, B, G	10
*pDR5::GFP/CUC2:VENUS* time-lapse and expression	Fig. 3D	3
*BLS::3xGFP* expression	Fig. S6 W	7

***A. thaliana*: NPA treatment (10 uM)**		

time-lapse	Fig. S3 A, C, E, G, I	3

***A. thaliana*: IAA treatment (5 um)**		

time-lapse	Fig. 2J,K; S3 K-N	4

***A. thaliana*: *cuc2-3* mutant**		

time-lapse	Fig. 2G-I; S4 B, C, J	3

***A. thaliana*: *pin1* mutant**		

time-lapse	Fig. S4 D-G	4

***A. thaliana*: *RCOg***		

time-lapse	Fig. 4 O,P; S6 A, C, E, G	3
time-lapse: protrusion	Fig. S2 G-I	3
*RCOg-VENUS* expression	Fig. 4 V	5
*pPIN1::PIN1:GFP* expression	Fig. S5 H	3
Primordia contribution (Growth alignment map)	Fig. 4 H	5

***A. thaliana*: *BLS::STM***		

time-lapse	Fig. 4 S,T; S6 I,K,M,O	5
protrusion time-lapse	Fig. S2 J-L	5
extra protrusion (compared to WT)	Fig. S6 Q	5/8
*pPIN1::PIN1:GFP* expression	Fig. S5 I	8
*BLS≫STM-VENUS* expression	Fig. 4 X	7
Primordia contribution (Growth alignment map)	Fig. 4 I	3

***A. thaliana*: *p35S::AtKRP2***		

time-lapse	Fig. 5 C-G	4

***A. thaliana*: *RCOg-V/BLS::STM***		

*RCOg-VENUS expression*	Fig. 6 Q	4

***C. hirsuta*: WT**		

time-lapse	Fig. 1 D, F, H-J; 2F; S1 B, D, F, P-S	3
time-lapse protrusion	Fig. S2 D-F	3
Sinus initiation adjacent to the midrib (time-lapse)	Fig. 1 P	3
Growth/Proliferation vs PD-position (growth alignment map)	Fig. 1 L, N	3
Additional protrusion in terminal leaflet	Fig. S1 K	3
Protrusion growth alignment map	Fig. S1 U	3
Primordium contribution (growth alignment map)	Fig. 4 J	3
*pPIN1::PIN1:GFP* expression	Fig. 2 B, E; S5 C, D, E, J	6
*RCOg-VENUS* expression	Fig. 4 U; 6 R	6
*pCHSTM::STM-VENUS* expression	Fig. 4 W	7
*pChSTM::3xVENUS* expression	Fig. S6 W	5

***C. hirsuta*: NPA treatment (10 uM)**		

time-lapse	Fig. S3 B, D, F, H, J	3

***C. hirsuta*: *pML1::PIN1:GFP***		

*pDR5::VENUS* expression and leaf morphology	Fig. S5 F	5

***C. hirsuta*: *rco* mutant**		

time-lapse	Fig. 4 M, N; S6 B, D, F, H	3
*pPIN1::PIN1:GFP* expression	Fig. S5 K	7
Primordia contribution (Growth alignment map)	Fig. 4 K	3

***C. hirsuta*: *stm* mutant**		

time-lapse	Fig. 4 Q,R; S6 J, L, N, P, R	5
*pPIN1::PIN1:GFP* expression	Fig. S5 L	6
Primordia contribution (Growth alignment map)	Fig. 4 L	3

***A. thaliana* vs *C. hirsuta***		

Stomata appear earlier in *A. thaliana* vs *C. hirsuta*	Fig. S1 E, F	4 from each species

## Supporting Citations

The following references appear in the Supplemental Information: Bassel et al., 2014, Crane et al., 2013, Hejnowicz and Romberger, 1984, Rivara and Inostroza, 1997, Rubio-Somoza et al., 2014, Scarpella et al., 2006, Smith et al., 2006.

## Supplemental Information

Methods S1. Computational Methods Supplement, Related to STAR Methods Descriptions of PIN quantification plug-in, regional primordium analysis and triangle-based protrusion morphospaces, as well as all computational models.

Video Abstract

## References

[bib1] Abley, K., Sauret-Gueto, S., Maree, A.F., and Coen, E. (2016). Formation of polarity convergences underlying shoot outgrowths. eLife 5, e18165. 10.7554/eLife.18165 PMC4969039 27478985

[bib2] Ali, O., Mirabet, V., Godin, C., and Traas, J. (2014). Physical models of plant development. Annu. Rev. Cell Dev. Biol. 30, 59-78. 10.1146/annurev-cellbio-101512-122410 25000996

[bib3] Alon, U. (2006). An Introduction to Systems Biology: Design Principles of Biological Circuits (Chapman & Hall/CRC).

[bib4] Alvarez, J.P., Furumizu, C., Efroni, I., Eshed, Y., and Bowman, J.L. (2016). Active suppression of a leaf meristem orchestrates determinate leaf growth. eLife 5, e15023. 10.7554/eLife.15023 PMC5096885 27710768

[bib5] Bar, M., and Ori, N. (2014). Leaf development and morphogenesis. Development 141, 4219-4230. 10.1242/dev.106195 25371359

[bib6] Barbier de Reuille, P., Routier-Kierzkowska, A.L., Kierzkowski, D., Bassel, G.W., Schupbach, T., Tauriello, G., Bajpai, N., Strauss, S., Weber, A., Kiss, A., et al. (2015). MorphoGraphX: A platform for quantifying morphogenesis in 4D. eLife 4, 05864. 10.7554/eLife.05864 PMC4421794 25946108

[bib7] Barkoulas, M., Hay, A., Kougioumoutzi, E., and Tsiantis, M. (2008). A developmental framework for dissected leaf formation in the Arabidopsis relative Cardamine hirsuta. Nat. Genet. 40, 1136-1141. 10.1038/ng.189 19165928

[bib8] Bassel, G.W., Stamm, P., Mosca, G., Barbier de Reuille, P., Gibbs, D.J., Winter, R., Janka, A., Holdsworth, M.J., and Smith, R.S. (2014). Mechanical constraints imposed by 3D cellular geometry and arrangement modulate growth patterns in the Arabidopsis embryo. Proc. Natl. Acad. Sci. USA 111, 8685-8690. 10.1073/pnas.1404616111 PMC4060677 24912195

[bib9] Bharathan, G., Goliber, T.E., Moore, C., Kessler, S., Pham, T., and Sinha, N.R. (2002). Homologies in leaf form inferred from KNOXI gene expression during development. Science 296, 1858-1860. 10.1126/science.1070343 12052958

[bib10] Bilsborough, G.D., Runions, A., Barkoulas, M., Jenkins, H.W., Hasson, A., Galinha, C., Laufs, P., Hay, A., Prusinkiewicz, P., and Tsiantis, M. (2011). Model for the regulation of Arabidopsis thaliana leaf margin development. Proc. Natl. Acad. Sci. USA 108, 3424-3429. 10.1073/pnas.1015162108 PMC3044365 21300866

[bib11] Blein, T., Pulido, A., Vialette-Guiraud, A., Nikovics, K., Morin, H., Hay, A., Johansen, I.E., Tsiantis, M., and Laufs, P. (2008). A conserved molecular framework for compound leaf development. Science 322, 1835-1839. 10.1126/science.1166168 19095941

[bib12] Bolduc, N., Yilmaz, A., Mejia-Guerra, M.K., Morohashi, K., O’Connor, D., Grotewold, E., and Hake, S. (2012). Unraveling the KNOTTED1 regulatory network in maize meristems. Genes Dev. 26, 1685-1690. 10.1101/gad.193433.112 PMC3418586 22855831

[bib13] Bringmann, M., and Bergmann, D.C. (2017). Tissue-wide Mechanical Forces Influence the Polarity of Stomatal Stem Cells in Arabidopsis. Curr. Biol. 27, 877-883. 10.1016/j.cub.2017.01.059 28285992

[bib14] Coen, E., and Rebocho, A.B. (2016). Resolving Conflicts: Modeling Genetic Control of Plant Morphogenesis. Dev. Cell 38, 579-583. 10.1016/j.devcel.2016.09.006 27676429

[bib15] Cotterell, J., Robert-Moreno, A., and Sharpe, J. (2015). A Local, Self-Organizing Reaction-Diffusion Model Can Explain Somite Patterning in Embryos. Cell Syst. 1, 257-269. 10.1016/j.cels.2015.10.002 27136055

[bib16] Crane, K., Weischedel, C., and Wardetzky, M. (2013). Geodesics in heat: A new approach to computing distance based on heat flow. ACM Trans. Graph. 32, 152.

[bib17] De Veylder, L., Beeckman, T., Beemster, G.T., Krols, L., Terras, F., Landrieu, I., van der Schueren, E., Maes, S., Naudts, M., and Inze, D. (2001). Functional analysis of cyclin-dependent kinase inhibitors of Arabidopsis. Plant Cell 13, 1653-1668. 10.1105/TPC.010087 PMC139548 11449057

[bib18] Donnelly, P.M., Bonetta, D., Tsukaya, H., Dengler, R.E., and Dengler, N.G. (1999). Cell cycling and cell enlargement in developing leaves of Arabidopsis. Dev. Biol. 215, 407-419. 10.1006/dbio.1999.9443 10545247

[bib19] Etournay, R., Merkel, M., Popović, M., Brandl, H., Dye, N.A., Aigouy, B., Salbreux, G., Eaton, S., and Julicher, F. (2016). TissueMiner: A multiscale analysis toolkit to quantify how cellular processes create tissue dynamics. eLife 5, e14334. 10.7554/eLife.14334 PMC4946903 27228153

[bib20] Fox, S., Southam, P., Pantin, F., Kennaway, R., Robinson, S., Castorina, G., Sanchez-Corrales, Y.E., Sablowski, R., Chan, J., Grieneisen, V., et al. (2018). Spatiotemporal coordination of cell division and growth during organ morphogenesis. PLoS Biol. 16, e2005952. 10.1371/journal.pbio.2005952 PMC6211367 30383040

[bib21] Friml, J., Vieten, A., Sauer, M., Weijers, D., Schwarz, H., Hamann, T., Offringa, R., and Jurgens, G. (2003). Efflux-dependent auxin gradients establish the apical-basal axis of Arabidopsis. Nature 426, 147-153. 10.1038/nature02085 14614497

[bib22] Gan, X., Hay, A., Kwantes, M., Haberer, G., Hallab, A., Ioio, R.D., Hofhuis, H., Pieper, B., Cartolano, M., Neumann, U., et al. (2016). The Cardamine hirsuta genome offers insight into the evolution of morphological diversity. Nat. Plants 2, 16167. 10.1038/nplants.2016.167 PMC8826541 27797353

[bib23] Hareven, D., Gutfinger, T., Parnis, A., Eshed, Y., and Lifschitz, E. (1996). The making of a compound leaf: genetic manipulation of leaf architecture in tomato. Cell 84, 735-744. 10.1016/s0092-8674(00)81051-x 8625411

[bib24] Hay, A., and Tsiantis, M. (2010). KNOX genes: versatile regulators of plant development and diversity. Development 137, 3153-3165. 10.1242/dev.030049 20823061

[bib25] Hay, A., Barkoulas, M., and Tsiantis, M. (2006). ASYMMETRIC LEAVES1 and auxin activities converge to repress BREVIPEDICELLUS expression and promote leaf development in Arabidopsis. Development 133, 3955-3961. 10.1242/dev.02545 16971475

[bib26] Heisler, M.G., Ohno, C., Das, P., Sieber, P., Reddy, G.V., Long, J.A., and Meyerowitz, E.M. (2005). Patterns of auxin transport and gene expression during primordium development revealed by live imaging of the Arabidopsis inflorescence meristem. Curr. Biol. 15, 1899-1911. 10.1016/j.cub.2005.09.052 16271866

[bib27] Hejnowicz, Z., and Romberger, J.A. (1984). Growth tensor of plant organs. J. Theor. Biol. 110, 93-114.

[bib28] Karwowski, R., and Prusinkiewicz, P. (2003). Design and Implementation of the L+C Modeling Language. Electron. Notes Theor. Comput. Sci. 86, 134-152.

[bib29] Kierzkowski, D., Lenhard, M., Smith, R., and Kuhlemeier, C. (2013). Interaction between meristem tissue layers controls phyllotaxis. Dev. Cell 26, 616-628. 10.1016/j.devcel.2013.08.017 24091013

[bib30] Kuchen, E.E., Fox, S., de Reuille, P.B., Kennaway, R., Bensmihen, S., Avondo, J., Calder, G.M., Southam, P., Robinson, S., Bangham, A., and Coen, E. (2012). Generation of leaf shape through early patterns of growth and tissue polarity. Science 335, 1092-1096. 10.1126/science.1214678 22383846

[bib31] Long, J.A., Moan, E.I., Medford, J.I., and Barton, M.K. (1996). A member of the KNOTTED class of homeodomain proteins encoded by the STM gene of Arabidopsis. Nature 379, 66-69. 10.1038/379066a0 8538741

[bib32] Mansfield, C., Newman, J.L., Olsson, T.S.G., Hartley, M., Chan, J., and Coen, E. (2018). Ectopic BASL Reveals Tissue Cell Polarity throughout Leaf Development in Arabidopsis thaliana. Curr. Biol. 28, 2638-2646.e4. 10.1016/j.cub.2018.06.019 PMC6109230 30100337

[bib33] Maugarny-Cales, A., Cortizo, M., Adroher, B., Borrega, N., Gonçalves, B., Brunoud, G., Vernoux, T., Arnaud, N., and Laufs, P. (2019). Dissecting the pathways coordinating patterning and growth by plant boundary domains. PLoS Genet. 15, e1007913. 10.1371/journal.pgen.1007913 PMC6363235 30677017

[bib34] Nikovics, K., Blein, T., Peaucelle, A., Ishida, T., Morin, H., Aida, M., and Laufs, P. (2006). The balance between the MIR164A and CUC2 genes controls leaf margin serration in Arabidopsis. Plant Cell 18, 2929-2945. 10.1105/tpc.106.045617 PMC1693934 17098808

[bib35] Poethig, R.S. (1987). Clonal analysis of cell lineage patterns in plant development. Am. J. Bot. 74, 581-594.

[bib36] Rast-Somssich, M.I., Broholm, S., Jenkins, H., Canales, C., Vlad, D., Kwantes, M., Bilsborough, G., Dello Ioio, R., Ewing, R.M., Laufs, P., et al. (2015). Alternate wiring of a KNOXI genetic network underlies differences in leaf development of A. thaliana and C. hirsuta. Genes Dev. 29, 2391-2404. 10.1101/gad.269050.115 PMC4691893 26588991

[bib37] Richardson, A., Rebocho, A.B., and Coen, E. (2016). Ectopic KNOX Expression Affects Plant Development by Altering Tissue Cell Polarity and Identity. Plant Cell 28, 2079-2096. 10.1105/tpc.16.00284 PMC5059799 27553356

[bib38] Rivara, M., and Inostroza, P. (1997). Using longest-side bisection techniques for the automatic refinement of delaunay triangulations. Int. J. Numer. Methods Eng. 40, 581-597.

[bib39] Rubio-Somoza, I., Zhou, C.M., Confraria, A., Martinho, C., von Born, P., Baena-Gonzalez, E., Wang, J.W., and Weigel, D. (2014). Temporal control of leaf complexity by miRNA-regulated licensing of protein complexes. Curr. Biol. 24, 2714-2719. 10.1016/j.cub.2014.09.058 25448000

[bib40] Runions, A., and Tsiantis, M. (2017). The shape of things to come: From typology to predictive models for leaf diversity. Am. J. Bot. 104, 1437-1441. 10.3732/ajb.1700251 29885230

[bib41] Runions, A., Tsiantis, M., and Prusinkiewicz, P. (2017). A common developmental program can produce diverse leaf shapes. New Phytol. 216, 401-418. 10.1111/nph.14449 PMC5638099 28248421

[bib42] Sapala, A., Runions, A., Routier-Kierzkowska, A.L., Das Gupta, M., Hong, L., Hofhuis, H., Verger, S., Mosca, G., Li, C.B., Hay, A., et al. (2018). Why plants make puzzle cells, and how their shape emerges. eLife 7, e32794. 10.7554/eLife.32794 PMC5841943 29482719

[bib43] Scarpella, E., Marcos, D., Friml, J., and Berleth, T. (2006). Control of leaf vascular patterning by polar auxin transport. Genes Dev. 20, 1015-1027. 10.1101/gad.1402406 PMC1472298 16618807

[bib44] Segonzac, C., Nimchuk, Z.L., Beck, M., Tarr, P.T., Robatzek, S., Meyerowitz, E.M., and Zipfel, C. (2012). The shoot apical meristem regulatory peptide CLV3 does not activate innate immunity. Plant Cell 24, 3186-3192. 10.1105/tpc.111.091264 PMC3462624 22923673

[bib45] Shani, E., Burko, Y., Ben-Yaakov, L., Berger, Y., Amsellem, Z., Goldshmidt, A., Sharon, E., and Ori, N. (2009). Stage-specific regulation of Solanum lycopersicum leaf maturation by class 1 KNOTTED1-LIKE HOMEOBOX proteins. Plant Cell 21, 3078-3092. 10.1105/tpc.109.068148 PMC2782295 19820191

[bib46] Smith, C., Prusinkiewicz, P., and Samavati, F. (2004). Local Specification of Surface Subdivision Algorithms. Lect. Notes Comput. Sci. 3062, 313-327.

[bib47] Smith, R.S., Guyomarc’h, S., Mandel, T., Reinhardt, D., Kuhlemeier, C., and Prusinkiewicz, P. (2006). A plausible model of phyllotaxis. Proc. Natl. Acad. Sci. USA 103, 1301-1306. 10.1073/pnas.0510457103 PMC1345713 16432192

[bib48] Vlad, D., Kierzkowski, D., Rast, M.I., Vuolo, F., Dello Ioio, R., Galinha, C., Gan, X., Hajheidari, M., Hay, A., Smith, R.S., et al. (2014). Leaf shape evolution through duplication, regulatory diversification, and loss of a homeobox gene. Science 343, 780-783. 10.1126/science.1248384 24531971

[bib49] Vuolo, F., Mentink, R.A., Hajheidari, M., Bailey, C.D., Filatov, D.A., and Tsiantis, M. (2016). Coupled enhancer and coding sequence evolution of a homeobox gene shaped leaf diversity. Genes Dev. 30, 2370-2375. 10.1101/gad.290684.116 PMC5131777 27852629

[bib50] Willis, L., Refahi, Y., Wightman, R., Landrein, B., Teles, J., Huang, K.C., Meyerowitz, E.M., and Jonsson, H. (2016). Cell size and growth regulation in the Arabidopsis thaliana apical stem cell niche. Proc. Natl. Acad. Sci. USA 113, E8238-E8246. 10.1073/pnas.1616768113 PMC5187701 27930326

[bib51] Zuniga, A. (2015). Next generation limb development and evolution: old questions, new perspectives. Development 142, 3810-3820. 10.1242/dev.125757 26577204

