# Supplementary material for: A Growth-Based Framework for Leaf Shape Development and Diversity
Source: Cell. 2019 May 30;177(6):1405–1418.e17. doi: 10.1016/j.cell.2019.05.011 (PMC6548024; doi:10.1016/j.cell.2019.05.011)
Supplement: Methods S1. Computational Methods Supplement, Related to STAR Methods — Descriptions of PIN quantification plug-in, regional primordium analysis and triangle-based protrusion morphospaces, as well as all computational models. [file mmc1.pdf]

Cell, Volume *177*

Supplemental Information

A Growth-Based Framework for Leaf Shape Development and Diversity

Daniel Kierzkowski, Adam Runions, Francesco Vuolo, Sören Strauss, Rena Lymbouridou, Anne-Lise Routier-Kierzkowska, David Wilson-Sánchez, Hannah Jenke, Carla Galinha, Gabriella Mosca, Zhongjuan Zhang, Claudia Canales, Raffaele Dello Ioio, Peter Huijser, Richard S. Smith, and Miltos Tsiantis

Methods S1: Computational methods supplement

May 17, 2019

1 PIN1 quantification

PIN1 membrane localization and polarity was computed for developing *A. thaliana* and *C. hirsuta* leaves (Fig. 2C,E and S5) in MorphoGraphX using PIN1:GFP signal from 3D confocal stacks. A 3D triangulated surface approximating the leaf epidermis was extracted and segmented into cells prior to projecting PIN1:GFP signal on the surface, as described in Barbier de Reuille et al. (2015). This triangulation was used as input for MorphoGraphX plugins calculating the cellular axis of PIN1 polarity and the localization of PIN1 at each cell wall. For each cell wall, PIN1 localization was determined as described in Barbier de Reuille et al. 2015. To determine the cellular axis of PIN1 polarity we estimate the orientation of PIN1 signal in each cell as described below. We do not attempt to infer the direction of PIN1 polarity (i.e. a vector), as this requires resolving the side of the cell wall from which PIN1::GFP signal originates, and necessitates resolutions beyond what can be achieved with a confocal microscope.

The axis of cellular polarity was computed by separating the triangles of each cell into those near the cell boundary (within $1\mu m$) and those in the cell interior (the remaining triangles). The cellular background intensity of PIN1:GFP was obtained by computing S_I, the average signal intensity per μm^2 in the cell interior. For each border triangle T the intensity difference between S_T, the average signal intensity per μm^2 of T, and the background intensity S_I was computed

$$\Delta S_T = S_T - S_I, \tag{1}$$

along with the vector

$$\Delta P_T = P_T - P_C, \tag{2}$$

from the cell centroid P_C to P_T, the centroid of T. The following vector, capturing both the direction and intensity of PIN1 polarity for each triangle, was calculated

$$v_T = \Delta S_T \frac{\Delta P_T}{\|\Delta P_T\|}, \tag{3}$$

and binned based on the angle of P_T in the tangent plane of the surface (24 bins). For each bin i, centred on θ_i in the tangent plane, the average vector v_{θ_i} was computed, and used to estimate the orientation of cellular polarity by performing Pincipal Component Analysis (PCA) on all such vectors. Performing PCA on v_{θ_i} reduces the influence of cell geometry on orientation compared to the case when v_T are used directly. For example, using v_T in elongated rectangular cells biases orientations towards the short axis of the cell due to the relatively large number of samples on the long edges of the cell. To account for overall differences in signal between samples, the covariance matrix used to perform PCA was normalized using the average signal intensity of all triangles in the mesh. We use the first principal component to approximate the cellular axis of PIN1-polarization. The degree of PIN1 polarization along this axis was calculated by taking the difference between the eigen-values associated with the first and second principal components.

2 Regional analysis and growth alignment graphs

Regional analysis was performed on time-lapse data of growing leaves between 3-7 DAI in *A. thaliana* wild type, *RCOg* and *BLS::STM* transgenic backgrounds, as well as *C. hirsuta* wild type and *rco* mutant

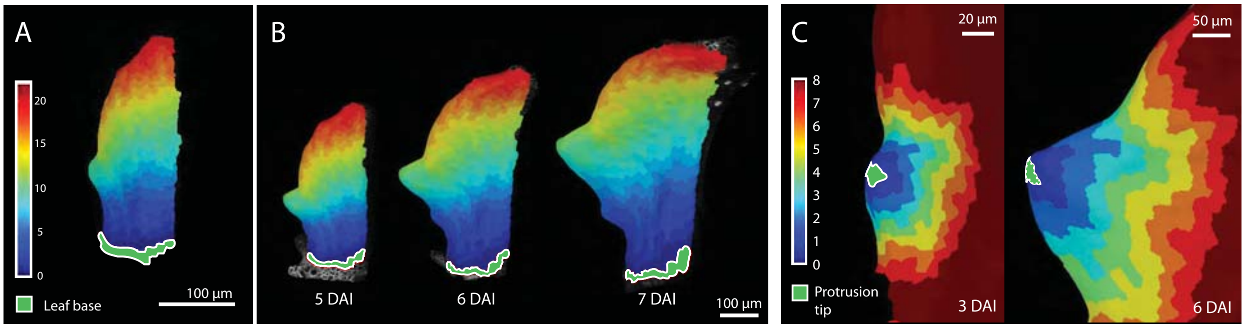

Display item 1: Regional analysis of primordia. (A-B) Regions of the primordium are identified based on the distance measured in cell-number from the leaf base (outlined green region) at 3 DAI (A). Distances between cell centers measured in μm were also used. This distance is propagated by lineage to later time-points (B), allowing the contribution of regions along the PD-axis to be quantified. (C) To quantify regional behaviour at protrusions the distance from the protrusion tip is calculated at 3 DAI and propagated to later time points.

backgrounds, and 2 DAI onwards in the *C. hirsuta stm* mutant background. This time-period spans from the first visible signs of marginal patterning to the establishment of clear morphological divergence between wild-type *A. thaliana* and *C. hirsuta* leaves (Fig. 1). The regional contribution of young primordia to shape divergence was evaluated using distances from key morphological features at T_0, the first time-point used in the analysis (Display item. 1). Distances were obtained from MorphoGraphX using a plugin that calculates the distance of each cell from a selected group of cells using Dijkstra's algorithm (Display item 1 A,C; green regions outlined in white). Analysis was performed on one half of the abaxial side of the leaf (including the leaf margin).

Lineage tracing was used to map distances at T_0 to clones at later time-points (Display item 1 B). This distance was then used to calculate growth alignment graphs based on averages (Fig. 1K-N, Fig. S1T-U) or proportional contributions (Fig. 4G-L) of area extension and cell-division as described below. Histograms, means and standard-deviations were computed using a MorphoGraphX plug-in, exported as CSVs and plotted using Excel.

To quantify the cellular behaviour of primordia along the proximal-distal axis (Fig. 1K-N) distances were measured in μm. To compute average proliferation and area extension along the PD-axis (Fig. 1K-N) clones at each time-point were binned by distance from the leaf base at T_0 (10-bins) prior to computing the average for each bin. To compensate for size differences between primordia, distances for each primordium were normalized by the maximum distance (i.e. primordium length), producing a relative position along the PD-axis. Quantifications of local behaviour at the first marginal protrusion (Fig. S1T-U) were performed using cellular-distance (i.e. the number of cells between each cell and the selected cell, Display item 1 C). Clones were binned by their cellular-distance (1-5 cells) from the protrusion tip at 3DAI.

Distance from the leaf base in μm was used to quantify the cellular contribution of regions along the PD-axis to primordium development (Fig. 4G-L, right). In this case, cells at T_0 were ordered based on their PD-position and divided into 10 bins, resulting in approximately the same number of cells in each bin. This allows the relative contribution of equivalently sized regions along the PD-axis to the leaf blade to be evaluated. From this, the cellular contribution of the cells in the ith bin was estimated using the formula

$$CC = \frac{C_{init}}{C_{clones}} C_{final} \quad (4)$$

where C_{init} was the number of cells in the bin at T_0, C_{final} was the number of cells in in the bin at the final time-point T_e, and C_{clones} was the number of clones present at T_e. This estimate corrects for cells present at T_0 which are not captured at all interleaving days of the time-lapse. The estimated cellular contributions were converted to percentages before plotting. The distal quartile of cells in Fig.4G-L (left panel, blue region), were identified by ordering cells based on their distance from a cell at the leaf base at T_0 (middle of the petiole) and marking the distal-most quartile.

3 Morphospace analysis of protrusion shape

Protrusion shape was quantified using a triangle approximating their overall form (Display item 2A). For developing *A. thaliana* and *C. hirsuta* leaves (Fig. 6S), the development of the first serration and leaflet (respectively) were tracked by identifying verticies coinciding with the tip, proximal and distal sinus of emerging protrusions in MorphoGraphX (Display item 2B). These vertices were identified in each primordium at 3 DAI, and chosen so as to coincide with cell wall junctions, which could be unambiguously traced in time-lapse data. The 3D positions of vertexes at successive time-points were used to form the triangle approximating the form of protrusions during development. For protrusions of mature leaves (Fig. 6T), the protrusions of at least 9 leaves of wild-type *C. hirsuta* and *A. thaliana* as well as transgenic *RCOg*, *BLS::STM* and *BLS::STM;RCOg* leaves in *A. thaliana* were measured in 2D using Fiji (second- and higher-order protrusions were ignored in *BLS::STM* and *BLS::STM;RCOg* leaves).

Measurements were imported into a custom program written in the L+C programming language (Karwowski and Prusinkiewicz, 2003) for visualization in LPFG. To obtain translation and rotation invariant measures, the edge lengths of the triangle were used to reconstruct all triangles in a common reference frame. The form of triangles was characterized by three parameters, extracted from their reconstructed forms (Display item 2B). These were the base width (length of the basal edge spanning the proximal to distal sinus), length (distance of the protrusion tip from the basal edge) and asymmetry (difference in length between the proximal and distal edges of the triangle). The height and width of a triangle captures the prominence of protrusions, whereas asymmetry captures proximo-distal asymmetries in form.

Together these parameters yield a 3-Dimensional translation and rotation-invariant shape-space (Display item 2 C-D). Figures in the main text (Fig. 6S-T) were generated by projecting data on axis-aligned slices through the 3D spaces depicted in Display item 2 (at base-width = 207 μm in Fig. 6S, base-width = 7.5 mm in Fig. 6T and asymmetry = 0 μm in Fig. 6S-T).

4 Model of leaf growth in the absence of marginal patterning

To simulate the convergent growth patterns observed in the absence of marginal patterning (Figs. 2G-K, S3, S4A-G) we devised a *default model* of leaf development (Fig. S4H-I). The model was implemented as a physically based model where growth was specified locally, and global changes in form emerged from a physically based simulation based on the Finite Element Method (FEM, c.f. Bassel et al. (2014); Kuchen et al. (2012)). Growth rates and directions were specified based on tissue type (blade, midrib and petiole) and differentiation. Due to the mechanical connections between neighboring regions, there can be differences between the growth specified and the resulting growth deformation computed using FEM. Following Kuchen et al. (2012) we call the former *specified growth* and the latter *resultant growth*.

The model was implemented in C++ using the VVE framework, which extends Vertex-Vertex systems (Smith et al., 2003) to provide specialized data-structures and utilities for the simulation of growing 2D tissues. The leaf was represented as a planar triangulation with an assigned uniform thickness covering the domain $\Omega \subset \Re^2$, with attributes stored at vertexes. Vertex attributes (e.g. position, tissue type and differentiation status) and connectivity were updated during each iteration of the simulation. To simulate physically based growth we used the FEM implementation described in Bassel et al. (2014) (see Section. 4.5 for details). The simulation loop for the model and additional details regarding its implementation are provided below. Simulation parameters are reported in Parameter table 1.

4.1 Simulation loop

Simulations were initialized with a mesh approximating a leaf primordium at 1 DAI with dimensions 107μm x 83μm (Display item 4A). Following initialization the simulation proceeds according to the execution loop depicted in Display item 3A. Each iteration starts by updating tissue identities (Sec. 4.2) and differentiation (Sec. 4.3). Next, growth rates and directions are determined (Sec. 4.4, Display item 3B), and used to update the rest configuration (i.e. unstressed configuration) of each triangular element (Display item 3C). The resulting growth deformation is determined by treating the leaf as a continuous isotropic Saint-Venant-Kirchhoff material and finding mechanical equilibrium using a semi-implicit Euler method (Display

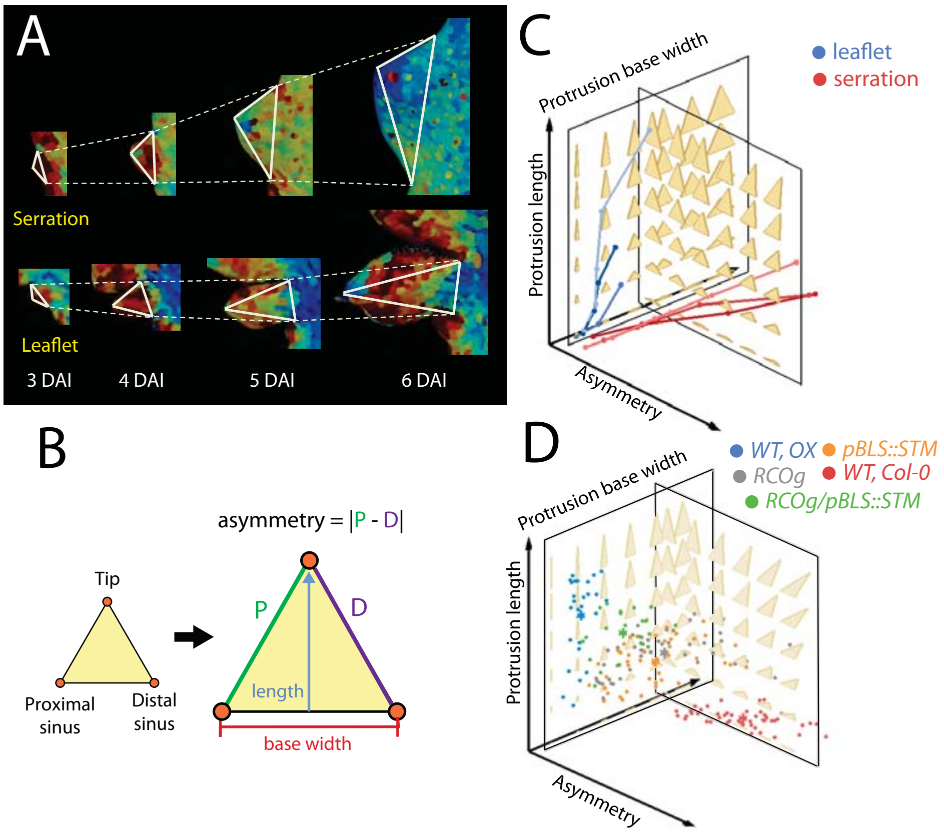

Display item 2: Protrusion morphospace. (A) Triangles spanning the proximal and distal sinuses and terminating at protrusion tips were identified at 3 DAI and traced based on lineage to subsequent time-points for the first serration of *A. thaliana* and first leaflet of *C. hirsuta* wild-type plants. (B) The 3D position of triangle vertices were used to compute triangle edge lengths, as well as the length, base width and asymmetry of protrusions. (C) The 3D morphospace parameterized by protrusion asymmetry, length and base width. Blue curves correspond to *C. hirsuta* leaflets ($n = 3$); red curves correspond to *A. thaliana* serrations ($n = 3$). (D) The 3D morphospace for mature marginal protrusions of wild-type *A. thaliana* (red, $n = 49$) and *C. hirsuta* (blue, $n = 26$), as well as those of the *A. thaliana* transgenics *RCOg* (grey, $n = 48$), *BLS::STM* (orange, $n = 45$) and *RCOg;BLS::STM* (green, $n = 21$). Planes intersecting the morphospaces show the triangular forms within those planes. The planes are placed at base width = 207 μm (C), 7.5 mm (D) (orthogonal to the asymmetry axis) and at asymmetry = 0 μm (orthogonal to the protrusion base width axis).

item. 3D; as in Bassel et al. (2014)). Once the resulting form is computed, residual stresses are released (as in Kuchen et al. (2012)). Finally the mesh is adaptively remeshed to improve mesh quality (Sec. 4.6).

4.2 Tissue-type specification

Before the petiole and midrib are established, the leaf is assumed to be a homogeneous primordium (Fig. S4H). The petiole and midrib regions are specified at a given time t_T. Both regions were determined geometrically based on positions along the proximo-distal and medio-lateral axes of the primoridum at the time t_T (PD and ML axis respectively, Display item 4).

Tissue-identities were determined on a per-vertex basis. Regions of the leaf less than $h_{petiole}$ from the leaf base were marked as petiole (Display item 4B). The midrib region was approximated using a triangle centered at the leaf base, extending h_{midrib} along the PD-axis and by w_{midrib} along the ML-axis (Display item 4C).

Following specification, tissue-identities were assumed to propagated by lineage. To maintain tissue identities following growth and remeshing, vertex positions at the time of midrib and petiole initiation t_T were stored and advected with mesh vertices during simulation. At later time-points of the simulation, regions comprising the midrib and petiole were determined on the basis of these coordinates.

4.3 Differentiation

Differentiation proceeds basipetally in both *A. thaliana* and *C. hirsuta*, progressing towards the leaf base. This progression coincides with a decrease in growth and proliferation (Figs. 1, S1). Similar to the model of leaf differentiation in *A. thaliana* proposed in Fox et al. (2018), we assume that differentiation is inhibited by a substance s which diffuses from the leaf base. Differentiation is assumed to occur when s drops below a threshold Th_{diff}. This captures the basipetal pattern of differentiation observed in both species.

To model the concentration of s in the 2D continuum Ω representing the leaf blade, we solve a diffusion-decay equation. As a simplification we assume that the time-scale of growth is slow relative to that of gradient establishment. Thus, s is the steady-state solution to the following PDE:

$$D\nabla^2 s - \rho s = 0, \tag{5}$$

where $s(X) : \Omega \to \Re$ maps each position in the leaf blade ($\Omega \subset \Re^2$) to a concentration, D is the rate of diffusion and ρ the turnover rate of s. To account for the introduction of s at the leaf base and diffusion in the blade, the leaf boundary $\delta\Omega$ is separated into two domains Γ_{base} and Γ_{margin} with different boundary conditions (Display item 4D). The boundary Γ_{base} coincides with the leaf/meristem boundary. Introduction of s at this boundary is captured using the Dirichlet boundary condition

$$s|_{\Gamma_{base}} = S_{base}, \tag{6}$$

which sets s to the concentration value S_{base} on Γ_{base}. For the leaf margin Γ_{margin}, we assume *no-flux* boundary conditions

$$\left.\frac{\partial s}{\partial n}\right|_{\Gamma_{margin}} = 0, \tag{7}$$

thus restricting diffusion to the blade.

To find the value of $s(X)$ at each vertex we discritize Eq.5 subject to the boundary conditions outlined in Eqs. 6-7 and solve the resulting system of equations. The Laplacian in Eq.5 is discritized using the discrete co-tangent Laplacian (as in Crane et al. (2013)), yielding a linear-system of equations

$$Av_s = b, \tag{8}$$

where A is the discritized version of Eq.5 (modified to account for boundary conditions), v_s is the vector of unknown concentration values (one for each vertex), and the RHS vector b takes a value of S_{base} for all vertices at the leaf base and 0 for all other vertices. We solve Eq. 8 using a graph-based Krylov method on the GPU (stabilized bi-conjugate gradient, see Bassel et al. (2014)).

To simulate the progression of differentiation we use the function $m(X) : \Omega \to \Re$, recording a maturity value for points in the blade (stored at mesh vertices). We assume that $s(X)$ locally inhibits maturation,

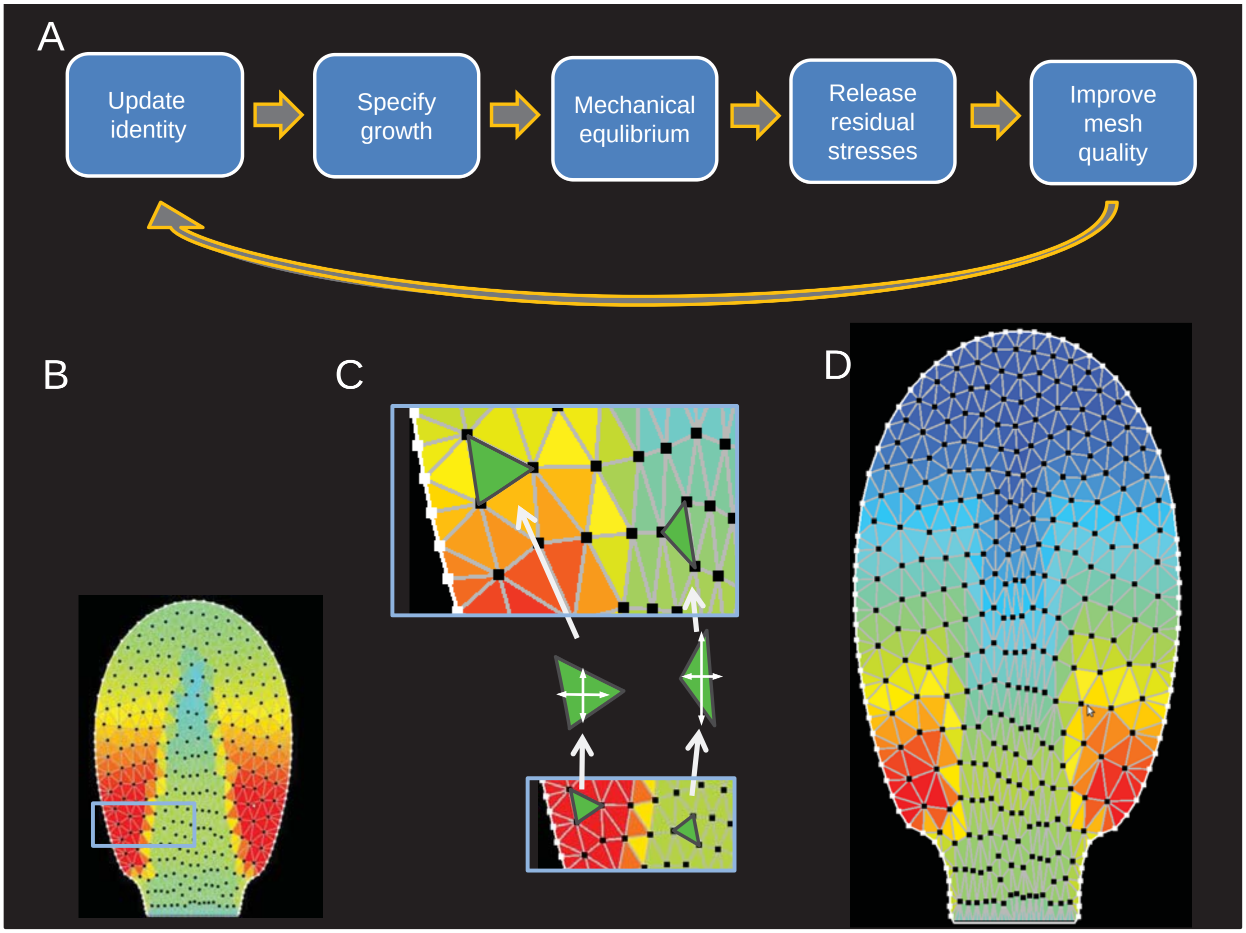

Display item 3: Default leaf model simulation loop. (A) Sub-phases of each simulation step, as described in Section 4.1. (B-D) Leaf and triangle geometry during an iteration of the simulation. The triangular mesh approximating a developing leaf with vertices (small squares; white on the boundary, and black in the leaf interior) and edges (grey lines) shown. Triangles are colored by the magnitude of specified growth. For visual clarity, mesh resolution is reduced in (B-D) compared to the simulations reported in the main text. (B) Specified growth is determined by differentiation and tissue type. (C) The rest configuration of each triangle is updated using its specified growth tensor, as depicted for two exemplar triangles shown in green (white crosses visualize growth tensors). (D) Global changes in leaf form are determined by finding mechanical equilibrium following growth. Residual stresses are released, and the next iteration then begins (specified growth for the next iteration is shown).

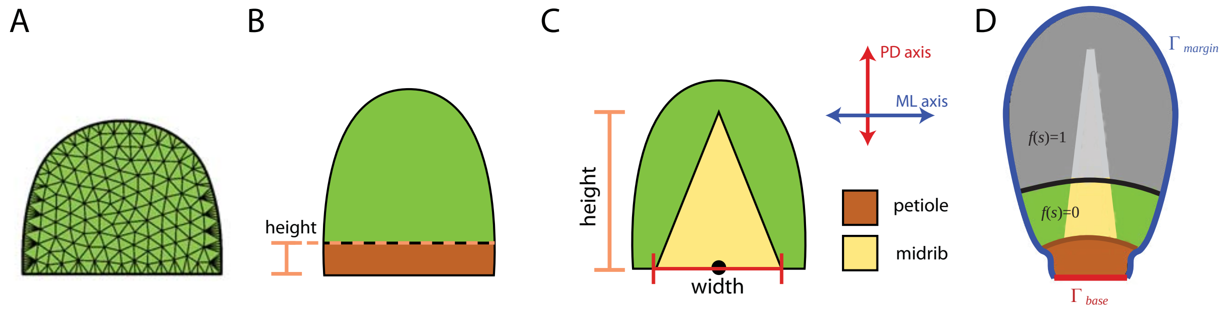

Display item 4: Initialization and regions pertinent to the description of the default leaf model. (A) Initial primordium shape with triangulation shown. (B) Portions of the leaf with height less than $h_{petiole}$ along the PD-axis are marked as petiole initials. (C) Portions of the leaf within the triangle at the leaf base of height h_{midrib} (PD-axis) and width w_{midrib} (ML-axis) are marked as midrib initials. (D) The simulation domain Ω has a boundary Γ which is separated into the leaf margin Γ_{margin} (blue) and base Γ_{base} (red). Differentiation begins when $s < Th_{diff}$ (black line marks $s = Th_{diff}$), making $f(s) = 1$.

and that differentiation begins when $m(X) = 1$. Prior to differentiation, maturation only increases when $s < Th_{diff}$. Following differentiation, maturation also increased at a regular rate, independent of $s(X)$, to capture the progression of differentiation. This leads to the following rate equation describing maturation of the blade

$$\frac{\partial m(X,t)}{\partial t} = M_s f_{[0,Th_{diff}]}(s) + M_d f_{[1,\infty)}(m(X)), \tag{9}$$

where M_s controls the rate of s-dependent maturation and M_d controls the rate of post-differentiation maturation, and $f_a(x)$ is the response curve used to relate these factors to maturation. For simplicity, we use a step-function $f_a(x)$ as our response curve, where $f_a(x) = 1$ for $x \in a$ and 0 outside this interval:

$$f(x) = \begin{cases} 1 & x \in a \\ 0 & otherwise \end{cases} \tag{10}$$

During each simulation step, the values of m are updated according to Eq. 9 and propagated by lineage following growth (i.e. advected).

4.4 Specified growth

The 2D growth of each point $X \in \Omega$ in the leaf blade is specified by the growth tensor $G(X)$, which in 2D can be represented by a symmetric 2×2 matrix (Hejnowicz and Romberger, 1984). The tensor $G(X)$ is specified using a component capturing isotropic growth ($G_{iso}(X)$), and one capturing anisotropic growth ($G_{aniso}(X)$):

$$G(X) = G_{iso}(X) + G_{aniso}(X), \tag{11}$$

The isotropic growth is

$$G_{iso}(X) = iso(X) boundary(X) I, \tag{12}$$

where I is a 2x2 identity matrix and $iso(X)$ is a scale factor that depends on tissue-type and differentiation status, and $boundary(X)$ captures growth inhibition at the leaf-meristem boundary (Eq. 14). In contrast, anisotropic growth is

$$G_{aniso}(X) = aniso(X) boundary(X) v(X) \otimes v(X), \tag{13}$$

where $aniso(X)$ is a scale factor that depends on tissue type and differentiation status, $v(X)$ is a 2D tissue-dependent vector ($\|v(X)\| = 1$) providing the direction of anisotropy ($\otimes$ denotes the tensor-product), and $boundary(X)$ is as in Eq. 12. This formulation specifically precludes the specification of local rotations, which are assumed to be an emergent aspect of growth. Growth inhibition at the leaf-meristem boundary is captured by

$$boundary(X) = \begin{cases} (1 - i_{boundary})^{\frac{b_{width} - y}{b_{width}}} & y \le b_{width} \\ 1 & y > b_{width} \end{cases}, \tag{14}$$

where y is the y-coordinate of X, $i_{boundary}$ is the proportion of growth inhibited in the boundary, and b_{width} is the width of the domain of inhibited growth at the leaf-meristem boundary.

Anisotropic growth (i.e. $aniso(X) > 0$) is assumed to occur throughout the early primordium (prior to 1.85 DAI), and in the midrib and petiole thereafter (after 1.85 DAI). The direction of anisotropic growth coincides with the PD-axis. In contrast, specified growth in the blade is assumed to be isotropic (i.e. $aniso(X) = 0$), although resultant growth may have an anisotropic component.

Each tissue type is assumed to grow at a different rate before and after differentiation. Observations indicate that following the onset of differentiation, proliferation decreases rapidly while growth decreases more gradually (Fig. 1 and S1). This is captured by the following equation:

$$diff(X) = \begin{cases} 0 & m(X) < 1 \\ \frac{m(X)-1}{d_{diff}} & d_{diff} > m(X) - 1 \geq 0 \\ 1 & m(X) - 1 \geq d_{diff} \end{cases} \tag{15}$$

which gradually decreases growth rates from their undifferentiated to differentiated values based on maturity $m(X)$ in the interval $[1, d_{diff} + 1]$, where d_{diff} controls the duration of this decrease following the onset of differentiation.

The equations for $iso(X)$ and $aniso(X)$ are then defined as

$$iso(X) = diff(X)i_{diff} + (1 - diff(X))i_{undiff} \tag{16}$$

$$aniso(X) = diff(X)a_{diff} + (1 - diff(X))a_{undiff} \tag{17}$$

where i_{undiff}, i_{diff}, a_{undiff} and a_{diff} are tissue dependent growth parameters (see Parameter table 1). Differentiation does not affect anisotropic growth during early development (prior to 1.85 DAI) which is specified by single scalar values indicating the anisotropic and isotropic growth components. In regions where midrib and petiole identities overlap, anisotropic growth occurs as in the petiole and isotropic as in the midrib.

4.5 Simulating growth

The rest-configuration for each triangle T, with vertices v_1, v_2 and v_3 is determined by its edge lengths l_1, l_2, l_3 (Bassel et al., 2014). The growth tensor is assumed to be constant over each triangle, and is determined by averaging the value of the growth-tensor function $G(X)$ (Eq. 11) at each vertex position

$$G_T = \frac{1}{3}(G(v_1) + G(v_2) + G(v_3)). \tag{18}$$

Releasing the residual stresses when mechanical equilibrium is achieved (Display item 3A), causes the rest and current configuration of the triangle to coincide. The rest-configuration following growth can thus be obtained by transforming the verticies based on the growth tensor (Display item 3C)

$$v_i^{t+\Delta t} = (I + (G_T)\Delta t)v_i, \tag{19}$$

where Δt is the duration of the growth step. Once transformed, the edge-lengths for T are calculated from the distances between $v_i^{t+\Delta t}$. As growth tensors are specified on a per triangle basis, common edges between neighboring triangles may have different lengths. In this case, we take the larger of the two rest-lengths as the edge length, which guarantees symplastic growth. These lengths provide an updated rest configuration for each triangle that incorporates the specified growth $G(X)$.

The deformation resulting from specified growth is then obtained using a mechanical simulation to compute the equilibrium following growth. The mechanical simulation treats each triangle as an isotropic two-dimensional hyperelastic element with the Saint Vernant-Kirchoff material law (Bassel et al., 2014). The degrees of freedom in the y-component of vertices on the leaf base Γ_{base} were fixed during simulation. Mechanical equilibrium was reached using a semi-implicit Euler method implemented on the graphics processing unit (GPU). This creates a sparse matrix problem (Bassel et al., 2014), which was solved using a graph-based Krylov method (stabilized bi-conjugate gradient). Once equilibrium is achieved, residual stresses are released (c.f. Kuchen et al. (2012)), by setting the edge lengths of triangles to their deformed length.

4.6 Remeshing

Releasing residual stresses causes the current and rest configurations to coincide, and allows for remeshing of the simulation domain. To increase the stability of numerical methods we update the mesh using variational remeshing to improve the quality of the triangle mesh (c.f. Runions et al. (2017), and references therein) via (i) edge-flipping, (ii) mesh-refinement and (iii) vertex-smoothing. Edge-flipping is performed as described in Runions et al. (2017). Mesh-refinement is performed by splitting triangles when their area or edge length exceeds a threshold, or when triangle *quality* ($Q(T)$, Eq. 20) drops below a threshold. Triangle quality is determined using a common measure of interpolation quality for linear triangular finite elements:

$$Q(T) = \frac{Area(T)}{(l_1 l_2 l_3)^{2/3}}, \tag{20}$$

where T is a triangle in the mesh with edge lengths l_1, l_2 and l_3. Triangles are split using the propagating bisection method proposed in Rivara and Inostroza (1997). Vertex-smoothing is performed by iteratively updating the position of interior vertexes to improve the quality measure $Q(T)$.

All persistent values used in simulations are located at mesh verticies, and are updated following refinement or smoothing of the mesh. Following refinement, values associated with vertices are initialized based on the barycentric coordinates of pre-existing vertices. Barycentric coordinates are also used to re-interpolate the values stored at vertices following smoothing.

5 Model of *Arabidopsis thaliana* leaf development

To model the development of a wild-type *Arabidopsis thaliana* leaf primordium we extended the preceding model (Section 4) to account for the dynamic growth patterns at the leaf margin that result from activity of the auxin-CUC2-PIN1 module (Fig. 1C,E, 2, S2). The activity of this module is integrated with differentiation and the mechanically based growth of the leaf blade (Sec. 5.3). Molecular processes are simulated in a file of cells representing the leaf margin using an extension of the patterning model proposed by Bilsborough et al. (2011) (Sec. 5.2), and these processes locally influence growth rates and differentiation in the blade. To determine the effects of auxin, CUC2 and PIN1 on growth and differentiation we rely on the time-lapse observations and PIN1 expression and orientation patterns reported in the main text (Figs. 1, 2, S1, S2, S5). Compared to the geometric growth model employed in Bilsborough et al. this model directly couples molecular processes at the margin to the differentiation and mechanically constrained growth of the tissue.

The simulation progresses according to the simulation loop in Display item 3, with two modifications. First, prior to computing the specified growth we update the geometry and molecular state of cells at the leaf margin (Sec 5.2). Second, the molecular state of margin cells influences the differentiation status and specified growth rates of the leaf blade (Sec 5.3). Additionally, we do not assume an anisotropic growth phase preceding blade initiation. To account for early patterns of anisotropy, we instead rely on the anisotropic growth induced by the auxin maximum forming at the leaf tip (which locally polarizes growth). Finally, simulations commence at 1.6 DAI once the proximal CUC2 domain is established. Aside from these modifications, the model is as described in Section 4. Parameters for the simulation shown in Fig. 3B are provided in Parameter table 2.

5.1 Leaf margin representation

The leaf is represented as a planar triangulation approximating a continuum Ω (as in Section 4). The margin is represented by a 1D sequence of cells of uniform width that are elongated by growth and divide (Display item 5). Each cell i on the margin is associated with a portion of the leaf boundary Γ_{margin} denoted C_i. The molecular state of cell i is characterized by an auxin concentration c_i, a CUC2 concentration $[CUC_i]$, the total amount of PIN1 in the cell $[PIN_i]$, a concentration $[PIN_{i\to j}]$ for each neighboring cell j recording the PIN1 concentration on the membrane of cell i facing cell j, and a differentiation value $diff_i$. The value of $diff_i$ is obtained by averaging the differentiation of the material points it is attached to

$$diff_i = \frac{1}{|C_i|} \sum_{v \in C_i} \mathit{diff}_{margin}(v), \tag{21}$$

Parameter name	Symbol	Text reference	Description	Value
Initialization				
Primordium width		Sec. 4.1	Initial width	$108\mu m$
Primordium height		Sec. 4.1	Initial height	$83\mu m$
Tissue identities				
Petiole specification time	t_T	Sec. 4.2	Time at which the petiole is specified	1.85 DAI
Petiole length	$h_{petiole}$	Sec. 4.2	Length of the petiole at specification	$24\mu m$
Midrib specification time	t_T	Sec. 4.2	Time at which the midrib is specified	1.85 DAI
Midrib length	h_{midrib}	Sec. 4.2	Distal extent of the midrib at specification	$134\mu m$
Midrib width	w_{midrib}	Sec. 4.2	Width of the midrib base at specification	$128\mu m$
Boundary width	b_{width}	Eq. 14	Width of inhibited region at the leaf-meristem boundary	$12\mu m$
Growth (specified)				
Primordium growth	a_{undiff}	Eq. 17	Rate of anisotropic primordium growth during initiation	$0.41d^{-1}$
	i_{undiff}	Eq. 16	Rate of isotropic primordium growth initiation	$0.35d^{-1}$
Blade growth	i_{undiff} (i_{diff})	Eq. 16	Rate of isotropic blade growth	0.56 (0.04) d^{-1}
Midrib growth	a_{undiff} (a_{diff})	Eq. 17	Rate of anisotropic midrib growth	0.51 $(0.045)d^{-1}$
Petiole growth	a_{undiff} (a_{diff})	Eq. 17	Rate of anisotropic petiole growth	0.28 $(0.09)d^{-1}$
	i_{undiff} (i_{diff})	Eq. 16	Rate of isotropic petiole growth	0.30 $(0.002)d^{-1}$
Boundary inhibition	$i_{boundary}$	Eq. 14	Proportion of growth inhibited at the leaf-meristem boundary	0.5
Differentiation				
Diffusion	D	Eq. 5	Diffusion rate for s	4.2 $\mu m^2 s^{-1}$
Decay	ρ	Eq. 5	Turnover rate for s	0.00073 s^{-1}
Threshold	Th_{diff}	Eq. 10	Threshold for s-dependent maturation inhibition	0.003 $mol\,\mu m^{-2}$
Leaf base concentration	S_{base}	Eq. 6	Concentration of s on Γ_{base}	1.0 $mol\,\mu m^{-2}$
s-dependent maturation	M_s	Eq. 9	Rate of s-dependent maturation (i.e. when $s < Th_{diff}$)	$1.17d^{-1}$
Post-differentiation maturation	M_d	Eq. 9	Rate of maturation following onset of differentiation	$1.17d^{-1}$
Growth decrease duration	d_{diff}	Eq. 15	Controls the duration of growth decrease following the onset of differentiation	3.5
FEM parameters				
Poisson's ratio			ratio of transverse to axial strain	0
Young's modulus			resistance to deformation	100
Thickness			Tissue depth (z-direction), assumed to be uniform	1
Visualization and numerics				
Growth time step	Δt	Eq. 19		$0.01d$
Anisotropy interval		Fig. S4I	Heatmap bounds for anisotropy	[1.0,3.0]
Area extension interval		Fig. S4I	Heatmap bounds for area extension	[0,300]

Parameter table 1: Parameters used in the default model of leaf development.

where $v \in C_i$ are the vertices of the triangulation in the interval C_i, and $|C_i|$ is the number of such verticies. The function $diff_{margin}$ determines the rate at which maturation of the blade affects molecular processes at the leaf margin. Consequently, $diff_{margin}$ has the same form as $diff(v)$ (Eq. 15) but uses d_{margin} in place of d_{diff}.

Physically based growth of the leaf deforms the marginal cells, and undifferentiated cells divide when their length exceed a length div_i. To capture auxin-dependent promotion of proliferation (Fig. S3 K and R), the division threshold for cell i depends on auxin concentration

$$div_i = \frac{Th_{div}}{1 + \kappa_{div} c_i}, \tag{22}$$

where Th_{div} is the basal size at which cells divide, and κ_{div} controls the rate at which auxin increases proliferation by reducing the division threshold. Following division daughter cells inherit the molecular state of their parent. Each cells i is assumed to differentiate with the adjoining region of the margin C_i, and stops dividing when $diff(v) > 1$ for all $v \in C_i$.

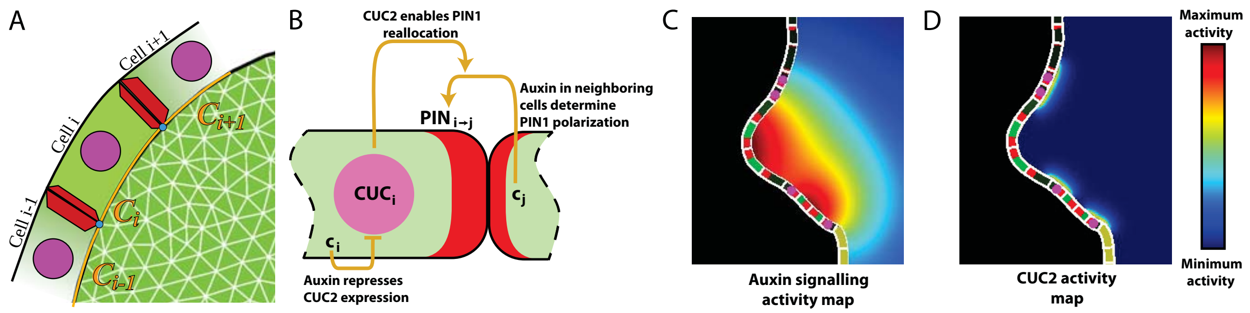

Display item 5: Leaf margin representation. (A) Three consecutive margin cells. Each cell i stores a concentration of CUC2 (pink circle), auxin (shade of green in the cell), a concentration of PIN1 on each membrane facing an adjacent cell (red wedges), and the total amount of PIN1 in the cell (not shown). The leaf boundary associated with cell i is C_i (adjacent segment of the orange curve bounded by blue circles). (B) Molecular interactions producing an interspersed pattern of auxin maxima and CUC2 expression proposed by Bilsborough et al. (2011). PINs are assumed to polarize towards neighboring cells with a high auxin concentration (i.e. up the gradient polarization). CUC2 enables PIN1 reallocation, but is repressed by auxin. Up-the-gradient polarization creates auxin maxima, whose positions are fixed by the subsequent down-regulation of CUC2. (C-D) Activity-maps in the leaf blade (C, auxin signalling; D, CUC2). The maps interpolate concentrations along the leaf margin, and extend these values into the blade.Values along the leaf boundary are calculated based on cellular concentrations, which are associated with the point on the boundary closest to the cell centre and linearly interpolated along the boundary between cell centers.

5.2 Marginal patterning

We simulate the molecular interactions determining the distribution of auxin, CUC2 and PIN1 at the leaf margin by extending the 1D model from Bilsborough et al. (2011) to incorporate differentiation. We use the molecular interactions from Bilsborough et al. as they lead to the organization of an alternating pattern of auxin maxima (with convergent PIN1 polarity towards the maxima) and CUC2 expression, as observed in confocal snapshots (Bilsborough et al., 2011) and time-lapse samples (Fig. 3D). We note, however, that the mechanistic basis of dynamic PIN1 polarization at the leaf margin and how this is influenced by CUC has yet to be resolved.

The dynamics of auxin, CUC2 and PIN1 are captured by the governing equations below. To drive margin simulations towards equilibrium, following each growth step the simulation of margin patterning is advanced $n_{chem} = 30000$ steps with a time-step of $\Delta t_{chem} = 0.05$ using forward-Euler integration. As in Bilsborough et al. (2011), we simplify equations by assuming that all cells have unit volume, and that cell-cell interfaces have unit area. For completeness, all equations are presented below, but for brevity we focus our description on aspects which have been modified in the present implementation and refer the reader to Bilsborough et al. (2011) for additional details.

The equations capturing the change of auxin concentration c_i in the ith cell are

$$\frac{dc_i}{dt} = \sigma((1 - diff_i)H - c_i) - \mu c_i - \sum_i \Phi_{i \to j} - \Phi_{int}. \tag{23}$$

The first term captures changes in auxin production, which approaches a target level controlled by H and differentiation $diff_i$, with a rate of σ. The assumed decrease in auxin production during differentiation accounts for the restriction of DR5 activity and PIN1 expression to proximal regions of the leaf (Alvarez et al., 2016; Hay et al., 2006). Auxin turnover is captured by the second term, and occurs with rate μ. The third term accounts for auxin fluxes between neighboring cells, where $\Phi_{i \to j}$ is the net-flux of auxin between cells i and j as captured by Eq. 25, described below. Finally, Φ_{int} captures the internalization of auxin at auxin maxima (Scarpella et al., 2006):

$$\Phi_{int} = T_{int}[c_i - c_{int}]_+, \tag{24}$$

which transports auxin to sub-epidermal layers at rate T_{int} when the auxin concentration c_i exceeds c_{int} (the function $[x]_+$ clamps to positive values). The net-flux of auxin between cells i and j, $\Phi_{i\to j}$, has the form

$$\Phi_{i\to j} = Tc_i[PIN_{i\to j}] - Tc_j[PIN_{j\to i}] + D(c_i - c_j). \tag{25}$$

Here, the first two terms capture PIN1 dependent auxin flux between cells i and j with T controlling the efficiency of transport. The final term captures apolar auxin flux which occurs at rate D, and represents diffusion through the plasmo-desmata as well as the action of apolar transporters. Note that $\Phi_{i\to j} = -\Phi_{j\to i}$, as a positive net-flux for i corresponds to a negative net-flux for j (and vice-versa).

Observations suggest spatio-temporal changes in the pattern of PIN1 expression during leaf development (Fig. 2A-B, S5). Auxin is believed to promote PIN1 expression (Smith et al., 2006), which is upregulated at sites of auxin accumulation. The preceding relations are captured by making he total amount of PIN1 in a cell $[PIN_i]$ a function of auxin concentration

$$[PIN_i] = (\rho_{IAA} IAA + \rho_{PIN}(1 - diff_i)), \tag{26}$$

where ρ_{IAA} controls auxin dependent expression, ρ_{PIN} accounts for auxin independent production which is eliminated by differentiation (Fig. 2A-B, Alvarez et al. (2016)).

The PIN1 proteins in each cell localize at the membrane, taking on a polar localization. When CUC2 is present PIN1 polarities at the leaf margin are consistent with up-the-gradient polarization (Bilsborough et al., 2011, and references therein), which posits that PIN1 proteins polarize based on the auxin concentration of neighboring cells. Accordingly, PIN1 in each cell is potentially allocated according to a variant of the formula proposed by Smith et al. (2006)

$$[PIN_{i\to j}]_{potential} = [PIN_i]\frac{b^{c_i}}{\sum_k b^{c_k} + d}, \tag{27}$$

This equation captures the quasi-steady-state localization of PIN1 when it is assumed to be allocated as an exponential function of auxin with base b, and deallocated at rate d. As PIN1 does not appear to re-polarize at the leaf margin in the absence of CUC2 (Bilsborough et al., 2011), we assume that PIN1 polarities become fixed when the concentration of CUC2 drops below a threshold Th_{CUC}. This assumption leads to the following equation for PIN1 concentration on the membrane of cell i facing cell j:

$$[PIN_{i\to j}] = \begin{cases} [PIN_{i\to j}]_{potential} & if [CUC_i] > Th_{CUC} \\ const & otherwise \end{cases} \tag{28}$$

According to this equation PIN1 proteins polarize up-the-gradient when CUC2 is present, but their localization becomes fixed when CUC2 expression is eliminated (i.e. $[CUC_i] < Th_{CUC}$). To account for changes in PIN1 membrane localization following differentiation, we use $(1 - \frac{diff_i}{c_i})[PIN_{i\to j}]$ as the membrane concentration when $diff_i > 0$. This accounts for the gradual elimination of PIN1 in differentiated regions of the leaf, as well as sustained PIN1 polarization at convergence points (Fig. S5) where auxin signalling is high.

The cellular concentration of CUC2 depends on auxin and differentiation. Following the onset of differentiation in cell i (when $diff_i > CUC_{diff}$) CUC2 expression is eliminated by setting $[CUC_i] = 0$. This accounts for the restriction of CUC2 expression to proximal regions of the leaf (Nikovics et al., 2006) and reduction of CUC2 activity by differentiation promoting TCP proteins (Rubio-Somoza et al., 2014). Otherwise, changes in CUC2 expression are captured by

$$\frac{d[CUC_i]}{dt} = \frac{\rho_{CUC}}{1 + \kappa_{CUC} c_i}[CUC_i] - (\nu + \nu_{IAA} c_i)[CUC_i]. \tag{29}$$

The production of CUC2 is captured by the first term, occurs with a base rate of ρ_{CUC}, and is inhibited by auxin at a rate of κ_{CUC}. The turn over of CUC2 is captured by the second term, occurs with a basal rate of ν but also depends on auxin with a rate of ν_{IAA}. Saturation of CUC2 production is captured by capping $[CUC_i]$ at a maximum value CUC_{MAX}. The auxin-dependent inhibition of CUC2 expression is consistent with mir164a dependent (ν_{IAA}) and independent (κ_{CUC}) regulation of CUC2 by auxin (Bilsborough et al., 2011; Nikovics et al., 2006). To guard against the spurious reintroduction of CUC2 following down-regulation by auxin, we set $[CUC_i] = 0$ when $[CUC_i] < 1e^{-10}$.

Auxin concentration at the boundaries (i.e. first and last cells) is assumed to be 0, reflecting low DR5 signal in this region following leaf initiation. Experimental observations indicate that the auxin-CUC2-PIN1 module is not active in the petiole where PIN1 expression and auxin signalling are not observed (Fig. 2A, S5A). To reflect these observations, following the establishment of the petiole CUC2 concentrations are set to 0 in this region and auxin production was eliminated by setting $H = 0$. Additionally, PIN1 concentration was set to 0 in all but the distal-most cells of the petiole on the left and right side of the margin, which were given a weak apical polarization ($0.25[PIN1_i]$).

5.3 Mapping marginal patterning to growth and differentiation

The influence of margin patterning is extended from the margin Γ_{margin} to the blade Ω by creating CUC2 and auxin-signalling activity maps $CUC(X) : \Omega \rightarrow \Re$ and $Auxin_S(X) : \Omega \rightarrow \Re$. To generate each map we use cellular concentrations to create boundary conditions for a diffusion-decay process. This allows marginal activity to be extended to the blade, but is not intended as a direct representation of the processes establishing these activity domains. We then use the activity maps to influence growth and differentiation of the blade. Conceptually, this is similar to the gradient based specification of hypothetical global polarizers and organizers in the growth polarized tissue framework (GFtbox) (Kuchen et al., 2012, and references therein). By contrast, we focus on accounting for the local activity of particular proteins and hormones, and the mapping of this activity to growth and differentiation.

The CUC2 activity map is obtained by solving

$$D_{CUC}\nabla^2 CUC(X) - \rho_{CUC} CUC(X) = 0, \tag{30}$$

subject to boundary conditions provided by the cellular model of the leaf margin. In this equation the combined effects of diffusion (with rate D_{CUC}) and turn-over (with rate ρ_{CUC}) localize CUC2 activity near the margin. To enforce consistency between the margin and adjoining blade we use Dirchelet boundary conditions, based on the cellular concentrations $[CUC_i]$. To this end, we associate each concentration with the cell centre (Display item 5), and linearly interpolate the values of adjacent cells to obtain a value for each point in Γ_{margin}. This yields a mapping $cuc(X) : \Gamma_{margin} \rightarrow \Re$, which is used to provides the boundary conditions

$$CUC(X)|_{\Gamma_{margin}} = \alpha_{CUC}[cuc(X)]_0^{max_{CUC}}, \tag{31}$$

where α_{CUC} controls the level of CUC2 activity in the blade, and the clamp function limits the effective range of CUC2 activity to $[0, max_{CUC}]$.

The auxin-signaling activity map $Auxin_S(X)$ is determined similarly, but is obtained by first calculating an auxin activity map $Auxin(X)$ which is used to define $Auxin_S(X)$. To obtain $Auxin(X)$ we solve the equation

$$D_A\nabla^2 Auxin(X) - \rho_A Auxin(X) = 0, \tag{32}$$

where the rates of diffusion D_A and decay ρ_A localize the effects of auxin. To extend cellular auxin concentrations c_i to the entire margin we linearly interpolate the values at cell centers to obtain $c(X) : \Gamma_{margin} \rightarrow \Re$ (Display item 5). To localize auxin activity near convergence points we use the following Dirchelet boundary conditions

$$Auxin(X)|_{c(X)\geq c_{min}} = c(X), \tag{33}$$

where c_{min} is the minimum concentration required to induce auxin activity. In the remainder of the margin (i.e. where $c(X) < c_{min}$) non-flux boundary conditions are assumed (as in Eq.7). This allows for the continuous extension of the values prescribed at maxima. The vertex values for $CUC(X)$ and $Auxin(X)$ are obtained by solving Eqs. 30 and 32 subject to their respective boundary conditions as described for $s(X)$ in Sec. 4.3. Finally, we convert the auxin activity map to a map of auxin signalling using the hill-function

$$Auxin_S(X) = \alpha_{auxin}\frac{Auxin(X)}{1 + \kappa_{auxin} Auxin(X)}, \tag{34}$$

where α_{auxin} controls the level of auxin signalling, and κ_{auxin} controls the rate at which signalling saturates.

PIN1 convergence points and associated auxin maxima are assumed to locally affect growth and differentiation (Figs. 1-2, S1-3). The influence of auxin signalling on maturation is accounted for modifying Eq. 9 to be

$$\frac{\partial m(X,t)}{\partial t} = M_s f_{[0,Th_{diff}]}(s) + M_d f_{[1,\infty)}(m(X)) + M_{auxin} Auxin_S(X) f_{[0,1]}(m(X)) \quad (35)$$

which introduces a third term dependent on $Auxin(X)$, where M_{auxin} determines the rate of auxin induced differentiation. Multiplying this term by $f_{[0,1]}(m(X))$ limits the effects of auxin on maturation to undifferentiated portions of the blade.

Auxin induced growth is accounted for using the following growth tensor

$$G_{auxin}(X) = [Auxin_S(X) - CUC(X)]_+ (AUXIN_{iso} I + AUXIN_{aniso} v_{auxin}(X) \otimes v_{auxin}(X))(1 - \mathit{diff}(X)), \quad (36)$$

where $AUXIN_{iso}$ and $AUXIN_{aniso}$ are respectively the rates of auxin induced isotropic and anisotropic growth, and $v_{auxin}(X)$ is the direction of auxin-dependent anisotropic growth. The rate of auxin induced growth is proportionally increased by $Auxin_S(X)$, and decreased by $CUC(X)$. As growth directions at PIN1 convergence points mirror PIN1 polarities, which take on a polarization up-the-gradient of auxin signaling, we assume that $v_{auxin}(X)$ is parallel to the gradient of $Auxin_S(X)$. Thus

$$v_{auxin}(X) = \frac{\nabla Auxin_S(X)}{\|\nabla Auxin_S(X)\|}. \quad (37)$$

The final term $(1 - \mathit{diff}(X))$ eliminates auxin-induced growth following differentiation.

Auxin dependent growth, and CUC2 dependent growth inhibition in the blade are accounted for in the following equation for specified growth

$$G(X) = (G_{iso}(X) + G_{aniso}(X))[1 - CUC_{blade} CUC(X)]_+ + G_{auxin}(X), \quad (38)$$

replacing Eq. 11 from the default model. This equation introduces an auxin dependent growth term $G_{auxin}(X)$, and $CUC(X)$ as a factor that inhibits both auxin dependent (see Eq. 36) and tissue-dependent growth. The rate of tissue-dependent growth inhibition is controlled by CUC_{blade}. In the midrib and petiole specified growth is still as described in Sec. 4.4.

5.4 Model initialization

The expression patterns of PIN1 and CUC2 at the margin are initialized to mirror those observed in primordia following emergence (Bilsborough et al., 2011). Cells in the basal i_{CUC} proportion of the primordium are initialized to express CUC2 at CUC_{MAX}. As simulations do not account for early stages of leaf initiation (0-1.6 DAI), we account for auxin induced maturation during this stage by adding M_{init} times the right-hand-side of Eq. 35 to $m(t)$ during the first simulation step.

6 A geometric model of margin development

To conceptualize the processes shaping marginal protrusions, we devised a simplified geometric model (Figs. 4AB, 6U). This model captures the key aspects of leaf margin morphogenesis using the minimal geometry required to represent leaflets with a supporting stalk. The geometric model is specified as an L-system using the L+C extension of the C++ programming language (Karwowski and Prusinkiewicz, 2003). We assume that leaf margin shape emerges from three interacting components (Runions and Tsiantis, 2017; Runions et al., 2017): positioning of the organ growth zone, a margin pattern generator and local growth repression and activation. In simulations, the x-axis coincides with the longitudinal- or PD-axis and the y-axis corresponds to the lateral- or ML-axis.

The organ growth zone is captured by a global basipetal growth gradient, as observed in *A. thaliana* and *C. hirsuta*. The margin pattern generator is captured by introducing convergence points into CUC2 expressing regions exceeding a given length, as occurs in cellular models of leaf margin development (Bilsborough et al., 2011). To restrict margin patterning to proximal regions, we assume that the ability to create convergence points in CUC2 expressing regions is quickly lost once it leaves a small undifferentiated region at the leaf base. Finally, local growth regulation is introduced by locally modifying displacements rates based on the location of convergence points and CUC2 (as in Runions et al. (2017)). Protrusion shape emerges from interactions between growth, patterning and differentiation.

6.1 Margin representation

The margin is represented as a string of modules corresponding to intervals of CUC2 expression, protrusion flanks and convergence points (CPs) (for module definitions and parameters see Parameter table 3). Each module stores information related to its developmental age and the geometry of the portion of the margin it represents:

1. CUC2 domains: The module $CUC(P_s, P_e, A)$ stores the positions of the first and last point defining the expression domain of CUC2 (P_s and P_e respectively), and $A : [0, L] \rightarrow \Re$ a mapping from each point in the interval to its developmental age (where L is the length of the CUC expressing interval).

2. Protrusion flanks: The module $Flank(P_s, P_p, P_d, P_e)$ stores the geometry of a protrusion flank. The first and last point defining the flank are P_s and P_e respectively. Two auxiliary points, P_p and P_d, are also stored to provide the minimal geometry required to represent the structure of leaflets with petiolules (as seen, for example, in *C. hirsuta*).

3. Convergence points: The module $CP(P, a)$ stores the position of the convergence point P, and a developmental age a.

Models are constructed to ensure that the positions defining two adjacent modules have a common point, thus guaranteeing the continuity of simulated margins. For example, given sequential modules

$$CUC(C_s, C_e, A)Flank(F_s, F_p, F_d, F_e), \tag{39}$$

we will have $C_e = F_s$.

6.2 Simulation loop

The model is initialized with an interval of CUC2 expression, representing the morphgenetically competent region of a developing leaf. This is captured by the following axiom:

$$Axiom : CUC(P_s^0, P_e^0, A_0()) \tag{40}$$

where $P_s^0 = (0, 0)$ and $P_e^0 = (1, 0)$ are initial positions of the interval endpoints, and $A(x) = 0$ provides the initial developmental age of the interval. Following initialization, each iteration of the model involves (i) updating the developmental age of the margin (ii) displacing the positions defining each segment to simulate growth and (iii) margin patterning.

6.3 Developmental Age

Both CUC expressing intervals and convergence points have a developmental age. The developmental age of CUC expressing margin segments $A(x)$ is used to determine their competence to produce convergence points. The value of $A(x) = 0$ for regions of the margin residing in the proximal undifferentiated region (less than Th_{diff} from the leaf base). Once growth displaces points on the margin from this region their developmental age increases at a constant rate, as captured by the L-system rule

$$CUC(P_s, P_e, A) \rightarrow CUC(P_s, P_e, A_n), \tag{41}$$

where $A_n : [0, L]$ is defined as:

$$A_n(x) = \begin{cases} A(x) & x + x_s \in Th_{diff} \\ A(x) + 1 & x + x_s \notin Th_{diff} \end{cases}, \tag{42}$$

where x_s is the x-coordinate of P_s. The mapping $A(x)$ is stored as an array of n_{age} samples. When the CUC2 domain is grown this array is advected and then resampled to maintain a uniform sampling. For convergence points, the developmental age a is a scalar that influences the outgrowth of CPs, and is updated by the rule

$$CP(P, a) \rightarrow CP(P, a + 1). \tag{43}$$

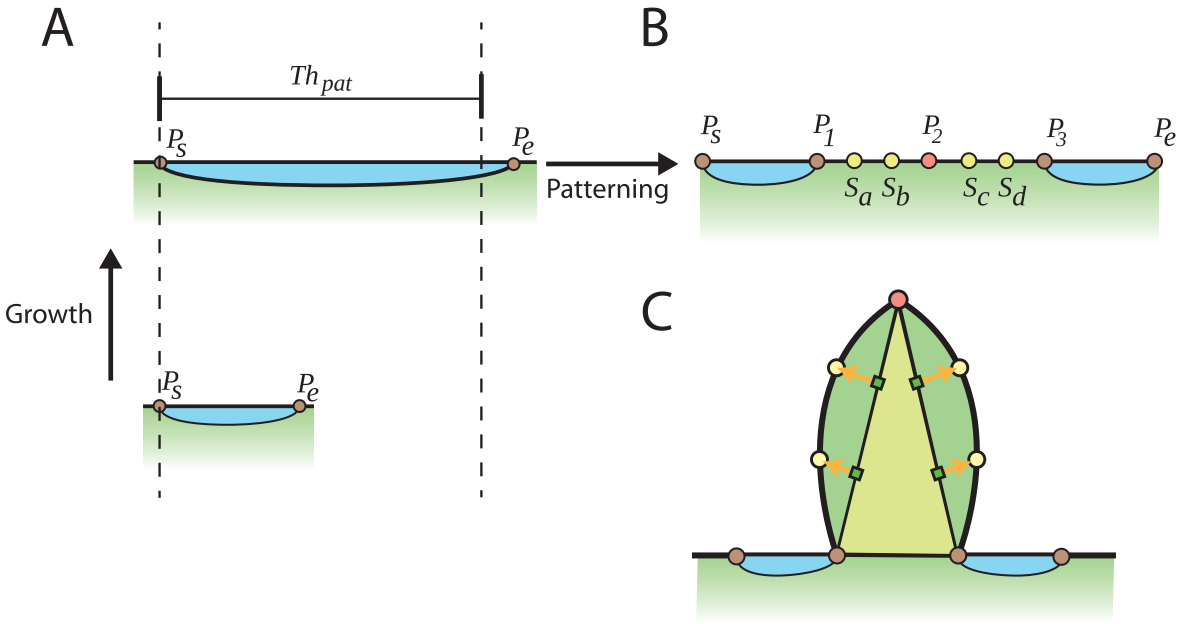

Display item 6: Geometric model of leaf margin development. (A) Growth increases the length of a CUC2 expression interval (blue interval spanning P_s to P_e). Patterning is triggered when $\|P_e - P_s\| > Th_{pat}$. (B) Patterning splits the CUC2 interval into four intervals of the same length, by introducing points P_1, P_2 and P_3. The first and last intervals correspond to $CUC()$ modules, whereas the second and third intervals correspond to $Flank()$ modules. A $CP()$ module (red disk) with position P_2 is placed between the two flank modules. The auxillary points (yellow disks) for each $Flank()$ module are uniformly spaced between the first and last position of the flank. (C) To move auxillary points in the direction of protrusion outgrowth, these points are displaced with reference points (green squares) placed uniformly along the edges of the triangle formed by P_1, P_2 and P_3. To account for growth in the other direction auxillary points are displaced orthogonal to the edge (orange arrows).

6.4 Patterning

The patterning of CPs in CUC2 intervals is driven by the growth dependent accumulation of space, and restricted to proximal regions by differentiation (Bilsborough et al., 2011; Runions et al., 2017). For a given module $CUC(P_s, P_e, A)$, the length L_{pat} provides the length of the undifferentiated sub-interval that can support patterning. This length is

$$L_{pat} = min\{\|P_s - P_e\|, \|P_s - P_c\|\}, \tag{44}$$

where P_c marks the PD position where competence is lost (i.e. where the developmental age A exceeds Th_{comp}). A new CP is introduced when L_{pat} exceeds the threshold length Th_{pat}, using a rewriting rule capturing the logic observed in time-lapse observations of *A. thaliana* leaf margin development (Fig. 3D). Accordingly, $CUC(P_s, P_e, A)$ is replaced with two smaller CUC domains separated by a CP and the proximal and distal flanks of a protrusion:

$$\begin{aligned} CUC(P_s, P_e, A) : \{L_{pat} > Th_{pat}\} \rightarrow CUC(P_s, P_1, A_1) Flank(P_1, S_a, S_b, P_2) \\ CP(P_2, a) \\ Flank(P_2, S_c, S_d, P_3) CUC(P_3, P_e, A_2). \end{aligned} \tag{45}$$

The positions P_1, S_a, S_b, P_2, S_c, S_d, and P_3 correspond to additional geometric samples of the margin introduced as depicted in Display item 6A-B. Finally, the developmental ages for the proximal and distal CUC expression domains (A_1 and A_2 respectively) are defined by the restriction of $A(x)$ to the corresponding intervals:

$$A_1(x) = A|_{[0,L_1]}(x), \tag{46}$$

where $L_1 = \|P_1 - P_s\|$

$$A_2(x) = A|_{[S,L]}(x + S), \tag{47}$$

where $S = L - L_2$ with $L_2 = \|P_e - P_3\|$. The developmental age of the CP is set to $a = A(L/2)$.

6.5 Growth

Growth of the margin is decomposed into two components. The first, accounting for longitudinal extension of the blade, is aligned with the x-axis. The second, accounting for lateral extension of the margin, is aligned with the y-axis.

Both components are affected by the global basipetal growth gradient. This is captured by the following relative elementary rate of growth which depends on the distance from the leaf base s:

$$RERG_G(s) = \begin{cases} 1 & s \leq Th_{gzs} \\ 1 - \frac{s - Th_{gzs}}{Th_{gze} - Th_{gzs}} & Th_{gzs} < s \leq Th_{gze} \\ 0 & otherwise \end{cases}. \tag{48}$$

where the coefficients Th_{gzs} and Th_{gze} determine the global shape of the growth gradient. Global growth begins to decrease at Th_{gzs} and is eliminated at Th_{gze}. The x-displacement of a point $P = (x, y)$ on the margin is obtained by integrating $a_s RERG_G(x)$ along the margin (i.e. x-axis), where a_s is the basal growth rate. To account for local growth inhibition by CUC2, in CUC intervals the value of $RERG_G(s)$ is multiplied by an inhibition factor $(1 - r_{CUC})$ prior to integration, where r_{CUC} controls the proportion of growth inhibited by CUC2.To capture local RCO dependent inhibition at protrusion bases, longitudinal displacement of protrusion flanks relative to their neighboring CP is reduced by the factor $(1 - r_{base})$, where r_{base} determines the proportion of displacement eliminated.

Lateral displacement at each convergence point $CP(P, a)$ is captured by the following equation, which depends on x position and the developmental age of the CP

$$\frac{dy}{dt} = (a_{lat} y + a_{cp}) RERG_x(x + a_{age} a), \tag{49}$$

where a_{lat} controls the rate of uniform lateral growth, and a_{cp} captures local growth at the protrusion tip due to auxin. Additionally, a_{age} controls the rate at which maturation of the CP reduces outgrowth of the

protrusion. This causes protrusion outgrowth to decrease along the PD-axis and with the developmental age of the CP.

Displacement rates for auxiliary points on the flanks of protrusions are assumed to follow the local growth directions of the protrusion. To capture growth in the direction of protrusion outgrowth these points are displaced with the reference points shown in Display item 6C (green squares). Displacement v in the orthogonal direction (orange arrows) captures auxin induced outgrowth of the margin and occurs at a rate of

$$\frac{dv}{dt} = a_{aux}(a_{lat}y + a_{cp})RERG_x(x + a_{age}a), \tag{50}$$

where $a_{aux} < 1$ controls the falloff of the outgrowth at auxiliary points. This rate coefficient decreases away from the convergence point, taking a value of a_{aux1} at the first and a_{aux2} at the second auxillary point ($a_{aux2} < a_{aux1}$). In Fig 6U, RCO dependent growth repression within the protrusion is simulated by reducing a_{aux2}.

Parameter name	Symbol	Text reference	Description	Value
Initialization				
Primordium width		Sec. 4.1	Initial width	$147\mu m$
Primordium height		Sec. 4.1	Initial height	$167\mu m$
Primordium CUC2 proportion	i_{CUC}	Sec. 5.4	Proximal proportion with CUC2 expression	0.6
Maturation factor	M_{init}	Sec. 5.4	Auxin-induced maturation at initiation	4.0
Tissue identities				
Petiole specification time	t_T	Sec. 4.2	Time at which the petiole is specified	3.0 DAI
Petiole length	$h_{petiole}$	Sec. 4.2	Length of the petiole at specification	$49\mu m$
Midrib specification time	t_T	Sec. 4.2	Time at which the midrib is specified	3.0 DAI
Midrib length	h_{midrib}	Sec. 4.2	Distal extent of the midrib at specification	$292\mu m$
Midrib width	w_{midrib}	Sec. 4.2	Width of the midrib base at specification	$180\mu m$
Boundary width	b_{width}	Eq. 14	Width of inhibited region at the leaf-meristem boundary	$6\mu m$
Growth (specified)				
Primordium growth	a_{undiff}	Eq. 17	Rate of anisotropic primordium growth during initiation	$0\ d^{-1}$
	i_{undiff}	Eq. 16	Rate of isotropic primordium growth during initiation	$0.31\ d^{-1}$
Blade growth	i_{undiff} (i_{diff})	Eq. 16	Rate of isotropic blade growth	$0.31\ (0.17)d^{-1}$
Midrib growth	a_{undiff} (a_{diff})	Eq. 17	Rate of anisotropic midrib growth	$0.28\ (0.14)d^{-1}$
Petiole growth	a_{undiff} (a_{diff})	Eq. 17	Rate of anisotropic petiole growth	$0.37(0.11)d^{-1}$
	i_{undiff} (i_{diff})	Eq. 16	Rate of isotropic petiole growth	$0.085(0.003)d^{-1}$
Auxin induced growth	$AUXIN_{aniso}$	Eq. 36	Rate of auxin induced anisotropic growth	$1.27\ d^{-1}$
	$AUXIN_{iso}$	Eq. 36	Rate of auxin induced isotropic growth	$0.707\ d^{-1}$
CUC inhibition of default growth	CUC_{blade}	Eq. 38	Coefficient controlling inhibition of blade growth by $CUC(X)$	10
Boundary inhibition	$i_{boundary}$	Eq. 14	Proportion of growth inhibited at the leaf-meristem boundary	0.025
Differentiation				
s Diffusion	D	Eq. 5	Diffusion rate for s	$20.4\ \mu m^2 s^{-1}$
s Decay	ρ	Eq. 5	Turnover rate for s	$0.000884\ s^{-1}$
s Threshold	Th_{diff}	Eq. 10	Threshold for s-dependent maturation inhibition	$0.03\ mol\mu m^{-2}$
Leaf base concentration	S_{base}	Eq. 6	Concentration of s on Γ_{base}	$1.0\ mol\mu m^{-2}$
s-dependent maturation	M_s	Eq. 35	Rate of s-dependent maturation (i.e. when $s < Th_{diff}$)	$1.41\ d^{-1}$
Post-differentiation maturation	M_d	Eq. 35	Rate of maturation following onset of differentiation	$1.41\ d^{-1}$
Auxin dependent maturation	M_{auxin}	Eq. 35	Rate of auxin-induced maturation	$0.42\ d^{-1}$
Growth decrease duration	d_{diff}	Eq. 15	Controls the duration of growth decrease following the onset of differentiation	4.5
Patterning elimination duration	d_{margin}	Eq. 21	Controls the elimination of margin patterning following the onset of differentiation	3.25
Margin patterning				
Cell Division threshold	Th_{div}	Eq. 22	Maximum threshold length for cell-division	$48\mu m$
Sensitivity of division to auxin	κ_{div}	Eq. 22	Controls auxin induced cell-division	0.025
Auxin production rate	σ	Eq. 23	Rate at which auxin approaches target concentration H	0.5
Target auxin concentration	H	Eq. 23	Target concentration for auxin production	10
Auxin turnover	μ	Eq. 23	Rate of auxin turnover	0.0051
Polar transport coefficient	T	Eq. 25	Efficiency of PIN1 transport	2.5
Diffusion coefficient	D	Eq. 25	Rate of non-directional auxin flux	2.5
Auxin internalization threshold	c_{int}	Eq. 24	Threshold concentration for auxin-flux to internal layers	9
Rate of auxin internalization	T_{int}	Eq. 24	Rate of auxin-flux to internal layers	4
Auxin dependent PIN1 production	ρ_{IAA}	Eq. 26	Proportional relation between auxin and PIN1	0.1
Basal PIN1 expression	ρ_{PIN}	Eq. 26	Minimum PIN1 expression	1.5
Exponentiation base for PIN1 polarization	b	Eq.27	Exponent used to determine PIN1 allocation	2
PIN1 deallocation rate	d	Eq.27	PIN1 endocytosis rate	0.01
CUC2 threshold for PIN1 polarization	Th_{CUC}	Eq. 28	Minimum CUC2 for PIN1 repolarization	0.1
CUC2 production rate	ρ_{CUC}	Eq. 29		63
Sensitivity of CUC2 down-regulation to auxin	κ_{CUC}	Eq. 29	Controls auxin-dependent inhibition of CUC2	1.7
CUC2 turnover rate	ν	Eq. 29		3.6
Auxin-dependent CUC2 degradation rate	ν_{IAA}	Eq. 29	Controls auxin-dependent turnover of CUC2	0.03
Maximum CUC2 concentration	CUC_{MAX}	Eq. 29		5
Differentiation threshold for CUC2 elimination	CUC_{diff}	Sec. 5.2	Controls when differentiation eliminates CUC2 expression	0.1
Activity maps				
Diffusion of CUC2 activity	D_{CUC}	Eq. 30	Diffusion and turnover determine the range	20.4
Turnover of CUC2 activity	ρ_{CUC}	Eq. 30	of CUC2 activity in the leaf blade	0.127
CUC2 activity conversion factor	α_{CUC}	Eq. 31		0.1
Maximum CUC2 activity	max_{CUC}	Eq. 31		2.05
Diffusion of auxin activity	D_A	Eq. 32	Diffusion and turnover determine the range	205.7
Turnover of auxin activity	ρ_A	Eq. 32	of auxin activity in the leaf blade	0.0141
Auxin activity conversion factor	α_{auxin}	Eq. 34		0.13
Saturation rate of auxin activity	κ_{auxin}	Eq. 34		0.416
Minimum auxin level	c_{min}	Eq. 33		7
FEM parameters				
Poisson's ratio			ratio of transverse to axial strain	0
Young's modulus			resistance to deformation	100
Thickness			Tissue depth (z-direction), assumed to be uniform	1
Visualization and numerics				
Anisotropy interval		Fig. 2B	Heatmap bounds for anisotropy	[1.0,3.0]
Area extension interval		Fig. 2B	Heatmap bounds for area extension	[0,300]
Growth time step	Δt	Eq. 19		$0.00625\ d$
Molecular processes time step	Δt_{chem}	Sec. 5.2		0.05
Molecular processes steps	n_{chem}	Sec. 5.2		30000

Parameter table 2: Parameters for wild-type *A. thaliana* leaf development simulation. As molecular processes at the margin and activity maps are driven towards their steady-state at each simulation step we provide unitless parameters for related parameters of the model.

L-system Modules		Variables
$CUC(P_s, P_e, A)$	P_s	start position (proximal)
	P_e	end position (distal)
	A	developmental age of each point on the interval
$Flank(P_s, P_p, P_d, P_e)$	P_s	start position (proximal)
	P_p	proximal sub-sample
	P_e	distal sub-sample
	P_e	end position (distal)
$CP(P, a)$	P	position
	a	developmental age of the CP

Parameter table 3: L-system modules and their parameters in the geometric model of leaf margin development.

	Figure	4B i	4B ii	4B iii	4B iv	4B v	4B vi	4B vii	6U	6U
Parameter	Description	WT *A. thaliana*	Decreased longitudinal growth	Increased longitudinal growth	Increased competence	Extended growth domain	Extended growth domain and decreased longitudinal growth	Inhibition at protrusion base	Sessile leaflets	Leaflets with petiolules
Th_{pat}	Threshold length for CP formation	20.5							30.5	—
$[Th_{gzs}, Th_{gze}]$	Organ growth zone falloff interval	$[400, 1500]$				$[800, 2500]$	—	—	—	—
Th_{diff}	Longitudinal position where differentiation begins	15								
Th_{comp}	Time till differentiation	4500			9500				6500	—
a_s	Base longitudinal growth rate	0.0002	0.00015	0.0004			0.00015	—	—	—
a_{lat}	Base lateral growth rate	0.0001							0.0001	—
a_{CP}	CP dependent growth rate	0.0035							0.003	—
a_{age}	Age dependent growth rate	0.02								
r_{CUC}	CUC growth repression	0.1								
r_{base}	Protrusion base growth repression	0						0.325	0.7	—
a_{aux1}	First auxiliary point displacement rate	0.25								
a_{aux2}	Second auxiliary point displacement rate	0.2								0.02
n_{age}	Number of age samples	60								

Parameter table 4: Parameters for the geometric model of leaf margin development. Unless otherwise indicated simulations use the default parameters provided for the WT *A. thaliana* simulation. Parameter values marked with – use the same value as the preceding row.
